# Tracking Energy Transfer across a Platinum Center

**DOI:** 10.1021/acs.jpca.2c02017

**Published:** 2022-07-26

**Authors:** Tammy X. Leong, Brenna K. Collins, Sourajit Dey Baksi, Robert T. Mackin, Artem Sribnyi, Alexander L. Burin, John A. Gladysz, Igor V. Rubtsov

**Affiliations:** † Department of Chemistry, Tulane University, New Orleans, Louisiana 70118, United States; ‡ Department of Chemistry, Texas A&M University, College Station, Texas 77842, United States

## Abstract

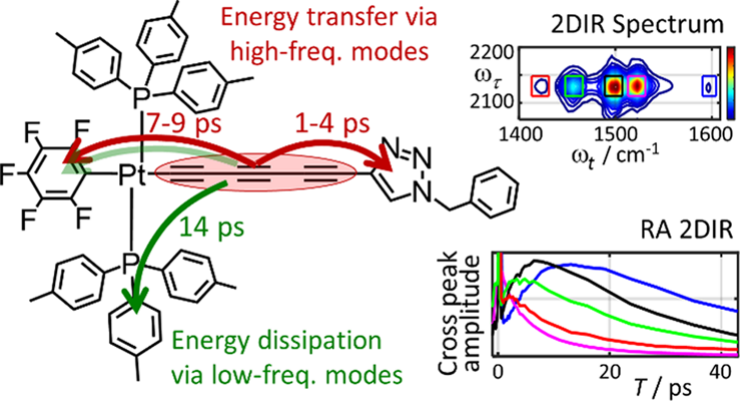

Rigid, conjugated alkyne bridges serve as important components in various transition-metal complexes used for energy conversion, charge separation, sensing, and molecular electronics. Alkyne stretching modes have potential for modulating charge separation in donor–bridge–acceptor compounds. Understanding the rules of energy relaxation and energy transfer across the metal center in such compounds can help optimize their electron transfer switching properties. We used relaxation-assisted two-dimensional infrared spectroscopy to track energy transfer across metal centers in platinum complexes featuring a triazole-terminated alkyne ligand of two or six carbons, a perfluorophenyl ligand, and two tri(*p*-tolyl)phosphine ligands. Comprehensive analyses of waiting-time dynamics for numerous cross and diagonal peaks were performed, focusing on coherent oscillation, energy transfer, and cooling parameters. These observables augmented with density functional theory computations of vibrational frequencies and anharmonic force constants enabled identification of different functional groups of the compounds. Computations of vibrational relaxation pathways and mode couplings were performed, and two regimes of intramolecular energy redistribution are described. One involves energy transfer between ligands via high-frequency modes; the transfer is efficient only if the modes involved are delocalized over both ligands. The energy transport pathways between the ligands are identified. Another regime involves redistribution via low-frequency delocalized modes, which does not lead to interligand energy transport.

## Introduction

For the past decade, compounds composed of long sp-hybridized carbon chains and transition-metal end groups have received substantial interest,^1−10^ leading to extensive research on the synthesis of transition-metal clusters containing alkynes and their derivatives.^11−13^ These ligands offer versatility in coordination modes with the possibility of donating electrons serving as promising candidates for novel materials for molecular electronics.^14−16^ Additionally, recent studies have indicated that these compounds could be useful as pharmaceuticals,^17^ optical wires,^18^ and electronic reservoirs.^19^

**Chart 1:**
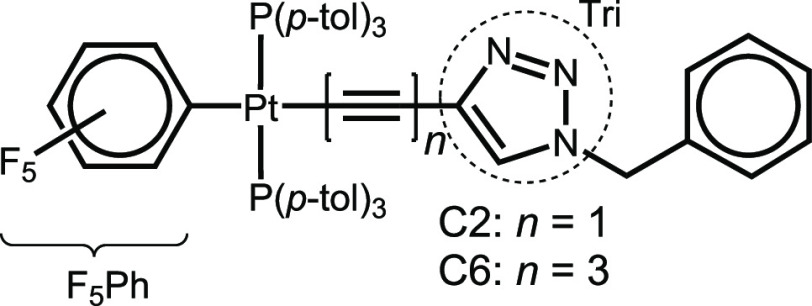
Structures of C2 (*n* = 1) and C6 (*n* = 3)

Linear and rigid alkyne-based molecular wires feature strong covalent bonds with frequencies in the convenient region of ca. 2100 cm^–1^. As such they can serve as useful infrared (IR) reporters for studying structures via two-dimensional infrared (2DIR) spectroscopy.^20^ Recent studies of ballistic transport of vibrational energy via oligomeric chains^21−23^ revealed that electronic conjugation within the chain can lead to higher transport speeds and ballistic transport efficiency.^24^ Alkyne-based molecular wires have not yet been tested as energy transporters. Here, we report on energy relaxation and transfer in two square planar Pt complexes featuring alkynyl triazole ligands with alkyne moieties of different lengths, C_2_ and C_6_, denoted as C2 and C6, respectively (Chart 1). These contain the F_5_Ph ligand opposite to the C_*n*_-Tri ligand and two trans tri(*p*-tolyl)phosphine ligands.

Transition-metal complexes and organic compounds with polyyne moieties (ligands) are widely used as bridges in donor–bridge–acceptor (DBA) compounds, providing conjugation enhanced electronic coupling of the electron donor and acceptor.^25^ Because of their unique properties of supporting conjugation and convenient vibrational frequency, such vibrationally excited bridges are attractive candidates for modulating electron transfer rates in DBA compounds.^26−28^ Vibrational relaxation dynamics of excited alkyne bridges are important for the electron transfer studies but not well understood.

Vibrational relaxation and thermalization in molecules occur via an intramolecular vibrational energy redistribution (IVR) process, which has been studied using a variety of experimental methods.^29−33^ Energy transfer and thermalization in covalent networks^34−38^ is better understood than the transfer across a metal center in a transition-metal complex for which only a few studies were reported.^39−42^ The coordination bonds at the metal center are often weak resulting in very inefficient energy transfer across the metal. For example, the lifetime of CO stretching modes in some metal carbonyls reaches 1 ns.^43−47^ A similar situation is encountered when an organic compound is attached to a metal or semiconductor surface. The surface binding energy is often small resulting in weak thermal conductivity through the interface.^48−52^ In contrast to surfaces, transition-metal complexes feature high-frequency vibrational modes on each side of the metal atom, which can potentially participate in the exchange of high-frequency quanta across the metal center. Understanding the role of energy transport involving high-frequency modes across the metal center is the objective of this study.

In this study, we use dual-frequency relaxation-assisted 2DIR (RA 2DIR) spectroscopy^53^ to investigate intramolecular energy redistribution and energy transport in the C2 and C6 compounds. This technique uses a pair of short mid-IR pulses to excite a vibrational tag at one end of the molecule, while a third mid-IR pulse probes various reporter modes in regions of the molecule spatially distant from the tag. When the excess energy arrives at the reporter site, it excites low-frequency modes coupled to the reporter thus enhancing the tag-reporter cross peak. The amplitude of the 2DIR cross peak between the tag and reporter changes with the time delay of the third pulse arrival, referred to as a waiting time, *T*, yielding energy transport kinetics.^53^

As detailed below, multiple 2DIR cross peaks of C2 and C6 demonstrated coherent oscillations. Vibrational and vibronic coherences have long been observed in polyatomic molecules in the ground^54,55^ and excited^56−59^ electronic states, respectively. In this study, we used coherent oscillations to identify modes located at the same moiety, which appears useful when severe peak overlap occurs in the linear Fourier transform infrared (FTIR) spectrum.

This paper is structured as follows: we start with assigning peaks in the FTIR absorption spectra of the two compounds using density functional theory (DFT)-based normal-mode analysis. Then the RA 2DIR data are discussed, reporting on the energy transport from and toward ν_C≡C_, as well as between different ligands. The experimental results highlighted include energy transfer times, cooling times, and frequencies of coherent oscillations observed for a range of various cross peaks. A computational section follows that clarifies the nature of energy transport pathways responsible for the observed RA 2DIR data. Special attention is given to identifying the requirements for efficient energy transfer across the Pt center.

## Experimental Details

### 2DIR Measurements

A detailed description of a fully automated dual-frequency three-pulse echo 2DIR instrument with heterodyne detection is presented elsewhere.^60,61^ Briefly, a Ti:sapphire laser operating at 1 kHz generates 80 fs pulses at 800 nm (Libra, Coherent) pumps a computer-controlled dual optical parametric amplifier (OPA, Palitra-duo, Quantronix). Each OPA output is directed to a computer-controlled noncollinear difference frequency generation unit (DFG; NIR Quantronix) to generate independently tunable mid-IR pulses used in the fully automated 2DIR instrument that features a sensitivity of better than 10^–4^ cm^–1^ in measured anharmonicities. The automatic frequency tuning to any diagonal or cross peak of choice within the range of 800–4000 cm^–1^ is achieved by using a direction stabilization schematic for each mid-IR beam. The spectral width of the mid-IR pulses was ∼150 cm^–1^, and the instrument response function, measured as a nonresonant signal, was fitted to a Gaussian function with the width σ_hwhm_ = 140 fs. 2DIR box-car measurements were achieved by scanning the delay between the first two mid-IR pulses τ at a fixed waiting time *T*, which is the delay between the second and third pulses, and recording the heterodyned spectrum in the frequency range of interest (λ → ω_*t*_) for every τ. Fourier transformation along τ results in the ω_τ_ (pump) axis in the 2DIR spectrum. For the RA 2DIR measurements, the 2DIR spectra were recorded for each waiting time, which was scanned with varying delay steps ranging from 100 fs at small waiting times up to 5 ps at large waiting times. It takes 1–2 h to acquire a waiting-time dependence with 40–50 points along *T*.

We used a dual-frequency RA 2DIR method^53^ to investigate energy relaxation and transport in the compounds. If a cross peak among spatially distant vibrational modes is measured, the cross-peak amplitude rises with the waiting time due to energy transfer from the mode initially excited by the first two laser pulses, the tag, to the vicinity of the reporter, the mode probed by the third laser pulse. The waiting time of the cross-peak maximum, *T*_max_, is referred to as the energy transfer time. A one-dimensional waiting-time trace for any diagonal or cross peak was obtained by integrating each 2DIR spectrum within a rectangular region centered at the peak. The waiting-time dependences were fitted with an asymmetric double sigmoidal function, , using Origin software. This function was selected because of its ability to fit the waiting-time data more accurately over longer waiting times than a bi-exponential function, accounting well for peak asymmetry. The reported *T*_max_ values were obtained by averaging five or more independent measurements.

### Sample Preparation

Compounds *trans*-(C_6_F_5_)(*p*-tol_3_P)_2_Pt(C≡C)_*n*_C=CHN(CH_2_C_6_H_5_)N=N with *n* = 1 and 3, referred to as C2 and C6 (Chart 1), were synthesized by the Gladysz group, as previously described.^3,7^ For FTIR and 2DIR measurements, ca. 15 mM CDCl_3_ solutions were used. The measurements were performed in a sample cell made of 1 mm-thick CaF_2_ windows and a 100 μm Teflon spacer at room temperature, 22 ± 0.5 °C.

### DFT Calculations and Vibrational Relaxation Modeling

Geometry optimization, normal-mode analysis, and anharmonic force constant calculations were performed using the Gaussian 09 suite. A B3LYP functional and a 6-311G(d,p) basis set were used for all elements except platinum. LANL2DZ basis sets and effective core potential were used for platinum atoms. Vibrational relaxation pathways of the end-group states were computed using a theoretical approach developed in refs (37, 38). The method uses DFT-computed anharmonic force constants of an isolated molecule to compute third-order relaxation pathways while the low-frequency modes of the molecule serve as a bath.^37,62^ The approach features a single variable parameter, cooling time to the solvent, which was set at 15 ps.^63−65^

## Results and Discussion

### Linear FTIR Spectroscopy Measurements

**Figure 1:**
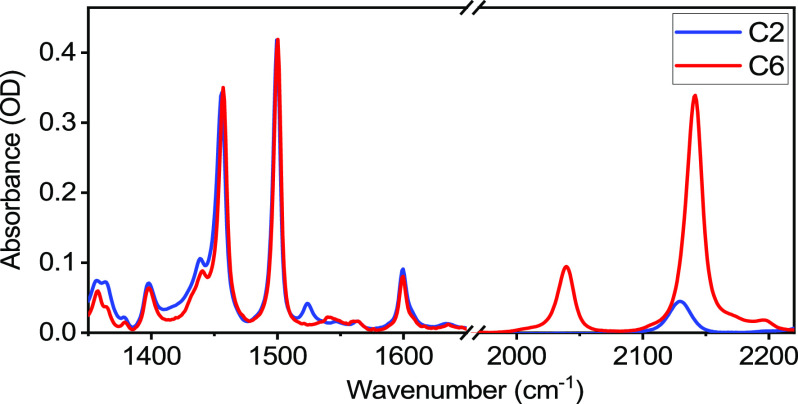
Solvent-subtracted infrared absorption spectra of compounds C2 and C6 in CDCl_3_. The spectrum of C6 was scaled by a factor of ca. 1.2 to match that of C2.

Linear absorption spectra of compounds C2 and C6 are very similar in the fingerprint region (Figure 1). Alkyne stretching peaks in C2 and C6 are different: there is a single peak at 2130 cm^–1^ for C2, while there are three peaks for C6, located at 2040, 2140, and ca. 2170 cm^–1^, originating from coupling of three C≡C local modes (Figure S1). The strongest peak for C6, found at 2140 cm^–1^, is due to in-phase motions of all three C≡C groups; unless stated otherwise, 2DIR measurements for C6 were performed for this peak, referred to as ν_C≡C_.

The spectra of both compounds in the fingerprint region feature intense peaks at 1460 and 1500 cm^–1^ and medium-strength peaks at 1600, 1435, and 1400 cm^–1^. The differences between C2 and C6 in the fingerprint region above 1350 cm^–1^ are minor, involving a peak at 1525 cm^–1^ for C2, while a similar peak in C6 is slightly weaker and observed at ca. 1539 cm^–1^, both referred to as a 1530 cm^–1^ peak (see the next section). Accurate peak assignment is paramount to understanding the results of the following 2DIR studies.

### DFT-Based Modeling of Linear Absorption Spectra

**Figure 2:**
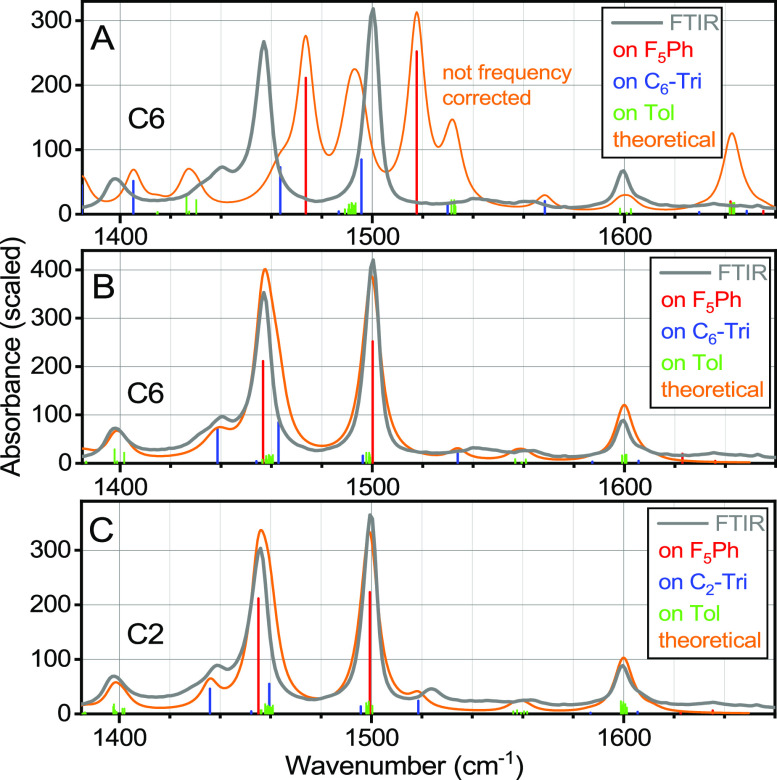
DFT-computed uncorrected line spectra for C6 (A) and frequency-corrected line spectra for C6 (B) and C2 (C). The modes associated with F_5_Ph-, C_*n*_-Tri, and Tol moieties are shown in red, blue, and green, respectively. Experimental linear absorption spectra for C6 (A, B) and C2 (C) in CDCl_3_ are shown with gray lines. Theoretical spectra of C2 (C) and C6 (B) obtained from applying a Lorentzian line shape with a full width at half maximum of 8.1 cm^–1^ are shown with orange lines. The *Y*-axes report the DFT-computed IR intensities (km/mole) for the line spectra. The experimental FTIR spectra in panels B and C (gray lines) were normalized to visually match the theoretical spectra.

To help assign peaks in the fingerprint region, we computed normal modes for both C2 and C6 compounds and constructed theoretical spectra by broadening the line spectra with a Lorentzian line shape of an area equal to the mode IR intensity. The computed line spectrum and theoretical (broadened) spectrum (orange line) for C6 are shown in Figure 2A. The amplitude of the lines in the line spectrum is equal to their computed IR intensities in km/mol, while the color of each bar reflects the location of the normal mode on F_5_Ph (red), C_*n*_-triazole-CH_2_Ph (C_*n*_-TriPh, blue), or six tolyl (Tol, green) moieties.

DFT-computed normal-mode frequencies for molecules in solution often appear higher than experimental values, mostly due to missing anharmonic corrections and solvent effects. As apparent from Figure 2A, uniform frequency corrections will not result in a good match with the experimental spectrum. However, the experimental spectrum is simple enough to implement different corrections to modes localized at different functional groups. For example, the two strongest modes, computed at 1517 and 1473 cm^–1^ (Figure 2A), reside at the F_5_Ph moiety and clearly correspond to the two strongest experimental absorption peaks at 1500 and 1460 cm^–1^, requiring a correction factor, κ, of 0.9885 (ν_corrected_ = κν). The same correction factor was applied to other modes of F_5_Ph. Corrections for tolyl groups were based on the peak at 1600 cm^–1^, which is characteristic of phenyl and Tol groups. A similar κ factor was applied to other modes located on Tol and C_*n*_-Tri moieties (see Table 1 footnote). The resulting frequency-corrected theoretical spectra for C6 and C2 (Figure 2B,C) show a good match with amplitude-scaled experimental spectra. Note that no attempt was made to tweak the correction factors within each group of modes to achieve a better match with the experiment.

**Table 1:** Main Experimental Absorption Peaks and DFT-Computed, Scaled Normal Modes of C2 and C6

	experimental peak, cm^–1^	computed peak,^a^ cm^–1^	IR intensity, km/mol	mode description (number of modes)
C2	1600	1599–1601	98 (23)^b^	Tol (6)
		1606	4	Tri (1)
		1622	3	F_5_Ph (1)
	1530^c^	1519	24	Tri (1)
	1500	1499	223	F_5_Ph (1)
		1498–1500	94 (22)^b^	Tol (6)
		1496	14	Tri (1)
	1460	1455	211	F_5_Ph (1)
		1456–1461	129 (18)^b^	CH_3_ of Tol (12)
		1459	55	Tri (1)
	1435	1436	46	Tri (1)
C6	1600	1599–1601	88 (18)^b^	Tol (6)
		1605	5	Tri (1)
		1623	20	F_5_Ph (1)
	1530^c^	1534	20	Tri (1)
	1500	1500	252	F_5_Ph (1)
		1498–1500	93 (22)^b^	Tol (6)
		1496	16	Tri (1)
	1460	1458	211	F_5_Ph (1)
		1456–1461	135 (17)^b^	CH_3_ of Tol (12)
		1463	85	Tri (1)
	1435	1434	73	Tri (1)

a Frequency correction factors were 0.9885 for all F_5_Ph modes and 0.978 and 0.974 for all Tri and Tol modes below and above 1550 cm^–1^, respectively.

b A sum of IR intensities of all modes in the range is given; the IR intensity of the mode with the largest IR intensity is given in parentheses.

c Actual experimental frequencies for the mode denoted as 1530 cm^–1^ differ for C2 (1525 cm^–1^) and C6 (1539 cm^–1^).

The modeling suggests that the peak at 1600 cm^–1^ is mostly due to Tol motion (Figure 2). The peaks at 1500 and 1460 cm^–1^ have dominant contributions from modes on F_5_Ph (see the SI for the way of computing the contributions). In addition, both peaks have significant (26–33%) contributions from Tol peaks, and the peak at 1460 cm^–1^ has a significant, 14% (20%) contribution from a single Tri mode for C2 (C6). The peak at ca. 1435 cm^–1^ is assigned to a Tri motion. As expected, the peaks at ca. 1530 cm^–1^ also belong to the alkyne-Tri moiety. The peak at 1400 cm^–1^ is assigned to Tol moieties.

The Tol modes contributing to the peaks at 1500 and 1600 cm^–1^ involve C–C stretching and C–H bending motions of the phenyl rings (Figure S3A,B), while CH_3_ bending modes of Tol moieties (Figure S3C) contribute to the peak at 1460 cm^–1^ (12 modes). The peak assignment made for C2 and C6 was further corroborated by the results of RA 2DIR measurements (see below).

The mode assignment is crucial for understanding the RA 2DIR data. Numerous cross peaks in 2DIR spectra were used to track the energy relaxation and transfer in the C2 and C6 compounds. The results are arranged into three groups involving energy transfer initiated by ν_C≡C_, energy transfer toward ν_C≡C_, and energy transfer initiated and detected by the modes in the fingerprint region.

### Energy Transfer Initiated by ν_C≡C_

**Figure 3:**
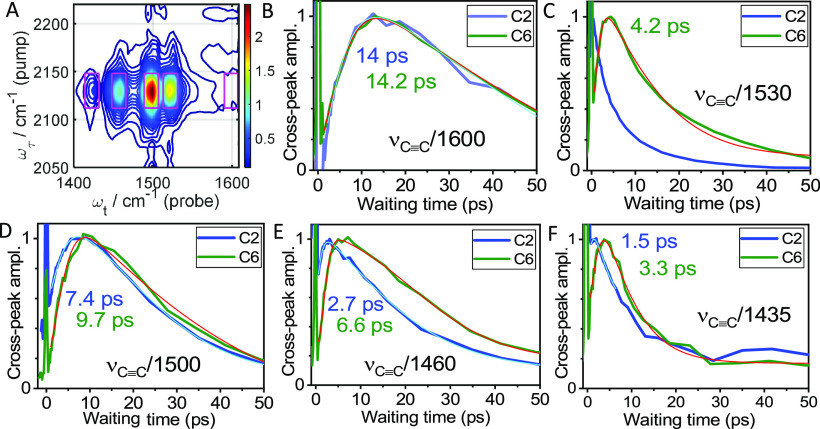
(A) 2DIR magnitude spectrum of C2 at *T* = 2.7 ps (see Figure S4 for C6). The magenta boxes show the integration windows for obtaining the waiting-time traces in panels (B–F). (B–F) Waiting-time traces for indicated cross peaks for C2 (blue lines) and C6 (green lines). The traces were fitted with an asymmetric double sigmoidal function (cyan lines for C2 and red for C6, see Experimental Details). *T*_max_ values are shown in the graphs with matching colors.

The first group of RA 2DIR experiments involves ν_C≡C_ as a tag and a variety of reporters shown with boxes in Figure 3A. The waiting-time dynamics for these cross peaks were recorded, characterizing energy transport from the excited ν_C≡C_ tag toward various reporter modes throughout the molecule. One-dimensional waiting-time traces of the cross-peak amplitude, constructed by integrating the cross-peak area within a respective box, are shown in Figure 3B–F. The *T*_max_ values, referred as the energy transport times, were obtained from the fits of the traces (see Experimental Details).^66^Table 2 summarizes the *T*_max_ values for the transport initiated by the ν_C≡C_ tag, which vary greatly for different reporters. Note that the ν_C≡C_ mode lifetimes were measured at 0.85 ± 0.1 and 1.9 ± 0.1 ps for C2 and C6, respectively (see Figure S2), which are much shorter than the measured *T*_max_ values for each compound.

**Table 2:** *T*_max_ Values Obtained from the Fit of the Waiting-Time Cross-Peak Traces for C2 and C6

cross peak	*T*_max_ (ps)		reporter location^a^
	**C2**	**C6**	
ν_C≡C_/1600	14	14.2	Tol
ν_C≡C_/1530	0	4.2	Tri
ν_C≡C_/1500	7.4	9.7	F_5_Ph, Tri, Tol
ν_C≡C_/1460	2.7	6.6	F_5_Ph, Tri, Tol
ν_C≡C_/1435	1.5	3.3	Tri

a Reporters contributing the most to the specific cross peak.

Most linear absorption peaks for both compounds have contributions of modes located at different moieties, Tri, F_5_Ph, and Tol. When such peaks serve as reporters, different contributors may feature different *T*_max_ values. Note that the cross-peak amplitude contributions of overlapping reporters scale as squares of their transition dipoles, or as their computed IR intensities (here we neglected the difference in the peak width). For the cross peaks connecting the spatially remote tag and reporter, the cross-peak amplitude also reflects the amount of excess energy delivered to the reporter, which is larger for reporters closer to the tag. As a result, a reporter mode, contributing less to the linear spectrum, can produce a stronger cross peak than a strong but remote reporter.

Despite severe reporter overlap in the linear spectrum, a good understanding of the energy transport pattern can be obtained from these experiments. For the ν_C≡C_ tag, short energy transport times are expected to the reporters at the triazole side as the two moieties are linked directly via a strong covalent bond. Indeed, the reporters with dominant Tri contributions (1435 and 1530 cm^–1^, Figure 3C,F) feature the shortest *T*_max_ values for C6 and dominating direct tag-reporter coupling for C2 (strongest cross peak at *T* = 0 ps). Interestingly, the tag-reporter (ν_C≡C_/Tri) coupling is much weaker for the C_6_ bridge, despite the tag delocalization over the three C≡C bonds.

The two strongest IR peaks at 1500 and 1460 cm^–1^, featuring the largest contribution from F_5_Ph (Table 1), show widely varying *T*_max_ values from 2.7 to 9.7 ps (Figure 3D,E). The transport times for the 1460 cm^–1^ peak are smaller than those for the 1500 cm^–1^ peak for both compounds due to a larger Tri contribution at 1460 cm^–1^ (Table 1). At the same time, the Tri mode contribution to the peak at 1500 cm^–1^ is small and does not affect the *T*_max_ significantly. Therefore, the F_5_Ph mode is the strongest contributor to the ν_C≡C_/1500 cm^–1^ cross peak. A contribution of Tol peaks to this cross peak could also be sizable but affect the cross peak only at larger delays. Note that the contribution of Tol modes to the ν_C≡C_/1460 cm^–1^ cross peak could be smaller than to ν_C≡C_/1500 cm^–1^ as the Tol modes within 1460 cm^–1^ peak reside further away at the Tol methyl groups, while the Tol modes at ∼1500 cm^–1^ reside at the phenyl rings. Therefore, the *T*_max_ for ν_C≡C_/1500 cm^–1^ cross peak is likely affected more by the Tol modes, resulting in their larger values. The 1600 cm^–1^ reporter is dominated by the Tol ring stretching modes, featuring the longest *T*_max_ values of 11.2 ps (C2) and 14.2 ps (C6), Figure 3B. These quantities are taken as characteristic energy transfer time from ν_C≡C_ to the Tol groups. To conclude, it takes approximately 1–4 ps to reach the triazole, 4–7 ps to reach F_5_Ph, and 11–14 ps to reach tolyls. Despite the complexity of the assignment, the transport times correlate with the tag-reporter distance, and all *T*_max_ times are longer for the C6 compound by ca. 3 ps, compared to C2.

A range of strongly coupled covalent bonds within the C_*n*_-Tri ligand offer efficient energy transfer pathways, in agreement with previous studies.^33,37,63,67^ Weak coordination bonds typically prevent efficient transfer across the metal atom.^40,68^ The energy transfer across the Pt center in C2 and C6 is investigated further computationally, *vide infra*.

Note that not all cross peaks for C2 exhibit a rise in their waiting-time traces. The waiting-time dependences for the ν_C≡C_/1530 and ν_C≡C_/1435 cross peaks show the largest amplitude at *T* = 0, although similar cross peaks for C6 show rises with *T*_max_ values of 4.2 and 3.3 ps, respectively (Figure 3C,F). The observation is not surprising as both 1435 and 1530 cm^–1^ modes are assigned to CC and CN stretching motions in the triazole ring, are spatially close to alkyne group, and are strongly coupled. Note that modes at the terminal phenyl ring of the C_*n*_-Tri ligand are weak and do not contribute much to the waiting time dependences.

### Energy Transfer toward ν_C≡C_

**Figure 4:**
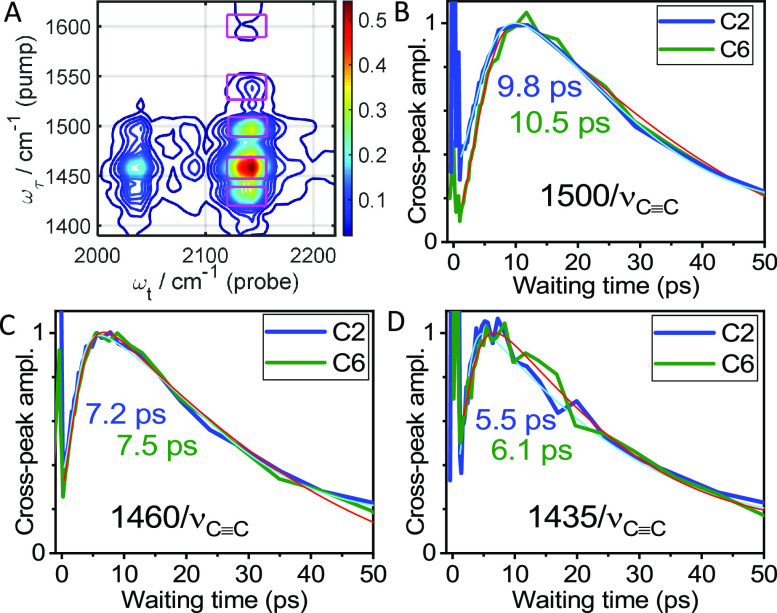
(A) 2DIR magnitude spectrum of C6 at *T* = 2.0 ps (see Figure S5 for C2). The magenta boxes show the integration windows for obtaining the waiting-time traces in panels (B–D). (B–D) Waiting-time traces for indicated cross peaks for C2 (blue lines) and C6 (green lines). The traces were fitted with an asymmetric double sigmoidal function (cyan lines for C2 and red lines for C6, see Experimental Details). *T*_max_ values are shown in the graphs with matching colors.

Relaxation pathways, resulting in energy transfer to remote moieties, are sensitive to the tag identity and location.^23,40^ Therefore, it is expected that reversing the tag and reporter would lead to different energy transfer dynamics, especially when high-frequency modes are involved in the pathways. Comparing the data for the reversed tag and reporter in each compound, the mechanism of energy transport can be assessed. In this section, we describe experiments where various peaks in the fingerprint region served as tags initiating energy transport while the ν_C≡C_ mode served as a reporter detecting energy arrival. Several cross peaks were measured (Figure 4A), and their waiting-time dependences were constructed (Figure 4B–D).

As expected, the two cross peaks with modes at triazole (1530 and 1435 cm^–1^) feature the shortest *T*_max_ values of ca. 5–6 ps (Figure 4D, Table 3). The direct coupling between the 1530 cm^–1^ mode and ν_C≡C_ is strong in C2 resulting in the highest cross-peak intensity at *T* = 0 (data not shown). The 1600/ν_C≡C_ cross peaks are too weak to record with confidence, as the excess energy escapes from the Tol moieties very slowly resulting in only a small amount arriving in the alkyne moiety region, see the next section. Similarly, other Tol modes do not contribute much to the 1500/ν_C≡C_ and 1460/ν_C≡C_ cross peaks. The 1500/ν_C≡C_ cross peak (Figure 4B) is dominated by the F_5_Ph contribution, as the Tri contribution to the 1500 cm^–1^ mode is small (<5%). The *T*_max_ values for this cross peak represent energy transfer to the F_5_Ph moiety. They are found to be only slightly larger (1–2 ps) than the *T*_max_ values for the reverse direction, ν_C≡C_/1500 (Tables 2 and 3). The *T*_max_ values for the 1460/ν_C≡C_ cross peaks are smaller than those for 1500/ν_C≡C_, explained by a stronger contribution of the Tri mode in the 1460 cm^–1^ peak. Convolution of two cross peaks, 1460(F_5_Ph)/ν_C≡C_ and 1460(Tri)/ν_C≡C_, results in smaller measured *T*_max_.

**Table 3:** *T*_max_ Values from the Waiting-Time Dependences of Several Cross Peaks of C2 and C6

cross peak	*T*_max_ (ps)		reporter location^a^
	C2	C6	
1600/ν_C≡C_	weak	weak	Tol
1530/ν_C≡C_	NA	5 ± 1.5	Tri
1500/ν_C≡C_	9.8 ± 0.2	10.5 ± 0.3	F_5_Ph, Tri, Tol
1460/ν_C≡C_	7.2 ± 0.2	7.5 ± 0.2	F_5_Ph, Tri, Tol
1435/ν_C≡C_	5.5 ± 0.5	6.1 ± 0.5	Tri

a Reporters contributing the most to specific cross peak.

### Diagonal Peaks and Cross Peaks in the Fingerprint Region

#### Diagonal Peaks

Diagonal 2DIR peaks were measured for all the main peaks in both compounds, and the waiting time traces were used to determine the lifetimes. Note that the diagonal 2DIR peaks for the absorption peaks with multiple overlapping contributions favor the strongest peak contributor as the diagonal-peak contribution is proportional to the fourth power of their transition dipole, while the contribution to the linear absorption spectrum scales with the square of the transition dipole.^69^ Therefore, the diagonal peaks at 1600 cm^–1^ are dominated by the modes of Tol, diagonal peaks at 1500 and 1460 cm^–1^ are dominated by the modes of F_5_Ph, and peaks at 1530 and 1435 cm^–1^ are dominated by the modes of C_*n*_-Tri. The traces were fitted with a double-exponential function, and the time constants, percent contribution of the fast component, and mean time decay are reported (Table 4). The fast decay time, *t*_1_, is assigned to the lifetime of the excited mode.

**Table 4:** Fit Parameters for Several Diagonal Peaks

	freq., cm^–1^	*t*_1_, ps	*t*_2_, ps	fast component contribution,^a^ %	*t*_mean_, ps	dominant contribution
C2	1600	1.00 ± 0.06	25 ± 2	56		Tol
C6	1600	1.06 ± 0.06	27 ± 2	55		
C2	1500	2.0 ± 0.1	9.1 ± 0.2	41	6.2	F_5_Ph
C6	1500	2.1 ± 0.1	9.5 ± 0.2	36	6.8	
C2	1460	1.93 ± 0.07	9.2 ± 0.3	52	5.4	
C6	1460	2.3 ± 0.2	9.6 ± 0.5	46	6.2	
C2	1530	1.8 ± 0.2	4.8 ± 0.9	71	2.6	C_2_-Tri
C2	1435	1.6 ± 0.2	5.9 ± 0.5	58	3.4	
C6^b^	1435	3.7 ± 0.1		100	3.7	C_6_-Tri

a Computed as *A*_1_/(*A*_1_ + *A*_2_) × 100%, where *A*_1_ and *A*_2_ are the amplitudes of the first and second exponential components, *t*_1_ and *t*_2_.

b The diagonal peak at 1530 cm^–1^ for C6 is too weak to measure with confidence.

The diagonal kinetic traces for the modes of the same moiety appeared to be similar in the overall shape (SI) and the lifetime of the modes tested. There is no surprise that the traces were similar for Tol moieties in both compounds as they spatially separated from the alkyne moiety introducing the only primary structure difference of the two compounds.

The modes of F_5_Ph, 1500 and 1460 cm^–1^, show similar but different kinetics. The lifetimes of both modes are ca. 2 ps in both compounds, and the cooling time is ca. 9.4 ps (Table 4). While the fast components for both modes lay within their error bars, the overall traces are systematically different (Figure S9), with longer mean decay times for C6 compared to C2. In addition, for each mode the decays are slower for C6. Nevertheless, the characteristic lifetime and cooling time provide a fingerprint of the F_5_Ph moiety in such compounds.

The two C_*n*_-Tri peaks at 1435 and 1530 cm^–1^ in C2 feature similar lifetimes of 1.7 ps and a similar cooling time of ca. 5 ps (Figures S10, S11). The mean decay times of both diagonal peaks in C2 are characteristically short at ca. 3 ps. The 1435 trace for C6 has a similar mean time of 3.7 ps but is essentially single exponential. The question remains if the 3.7 ps is the true lifetime of the 1435 cm^–1^ mode in C6 or the exponential behavior is a convolution of the lifetime and peculiar cooling dynamics.^70^ The diagonal peak at 1530 cm^–1^ for C6 is too weak to detect with confidence, indicating its small diagonal anharmonicity combined with weak IR intensity. Nevertheless, the kinetics of these peaks appear to be characteristic of the C_*n*_-Tri moiety.

**Figure 5:**
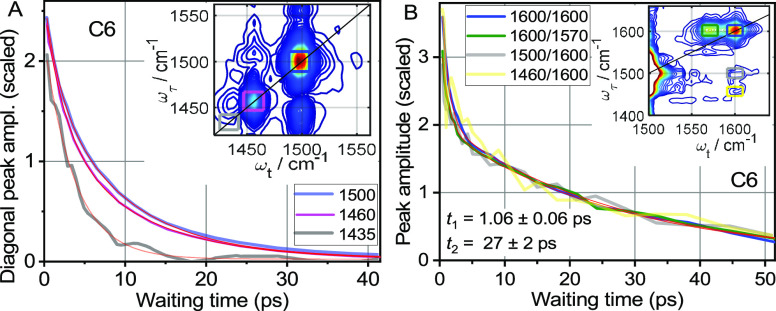
Scaled waiting-time traces for indicated diagonal and cross peaks for C6 (see Figure S6 for C2). (A) Results of individual fits of *T*-traces of diagonal peaks (red lines) are shown in Table 4. The insets in A and B show 2DIR spectra measured at 2.3 ps with color-matching boxes indicating the cross-peak integration regions. (B) 1600/1600 and 1600/1570 peaks were fitted globally with a double-exponential decay function (red lines) resulting in *t*_1_ = 1.06 ± 0.06 ps and *t*_2_ = 27 ± 2 ps and amplitudes of the fast component of 55% for 1600/1600 and 45% 1600/1570.

The diagonal-peak traces at 1600 cm^–1^ are very characteristic with the fast components of ca. 1.03 ps and slow components of ca. 26 ps (Figure 5B, Table 4, Figure S9A). Similar to other diagonal peaks, the slow component is attributed to the relaxation-assisted effect: lower-frequency modes coupled to the tag are excited via vibrational relaxation of the tag, thus perturbing the tag frequency and enhancing the diagonal peak. The slow component of the 1600 peak is slower than a typical cooling time of organic molecules to the solvent of ca. 15 ps^64^ and has a surprisingly larger amplitude *A*_2_ = 45% (Table 4). The large amplitude of the slow component is supported by the strength of the coupling among the modes of Tol, facilitated by its compactness and electronic conjugation.^24^ Slow cooling is caused by relative isolation of the Tol moieties from other functional group types and by closeness of three Tol groups within a single phosphine.

The long cooling time likely occurs due to relative isolation of the Tol moieties separated from the rest of the molecules by three dissimilar bonds involving heavy atoms, C–P, P–Pt, and Pt–C. The C–P–Pt–C bridge connecting Tol to F_5_Ph and C_*n*_-Tri features a small number of degrees of freedom and different low-frequency modes, so that mode delocalization across the bridge is limited making the energy transport inefficient, trapping the excess energy at tolyls. The closeness of three Tol groups within a single phosphine makes the cooling longer as cooling of 1600 cm^–1^ mode in one Tol moiety to another two Tol moieties of a phosphine group still contributes to the 1600 diagonal peak at later times.

#### Cross Peaks Involving 1600 cm^–1^ Mode

Two cross peaks involving 1600 cm^–1^ peak as a reporter (probed mode) and peaks at 1500 and 1460 cm^–1^ as tags (pumped modes) were also measured (Figure 5B, yellow and gray lines). Note that the main contributors for both tags reside at the F_5_Ph moiety. A rise of the amplitude is expected for a cross-peak between a tag at F_5_Ph and a reporter at Tol at delays exceeding 10 ps due to the significant distance between the moieties. However, no rise was observed. Instead, the waiting-time traces for both cross peaks follow the trace of the 1600 diagonal peak (Figure 5B) suggesting that both cross peaks originate fully from modes at the Tol moieties. Indeed, peaks at 1500 and 1460 cm^–1^ bear significant contributions from the modes at Tol (Figure 2, Table 1). The coupling of the Tol modes at 1500 and 1460 cm^–1^ (tags) and the Tol mode at 1600 cm^–1^ (reporter) is large, computed at ca. −3.8 cm^–1^, resulting in a significant cross-peak amplitude at *T* = 0. Relaxation of these tags populates lower-frequency modes in Tol similar to relaxation of the 1600 cm^–1^ tag, resulting in similar waiting-time traces for three different Tol tags at 1600, 1500, and 1460 cm^–1^. Apparently, the mode proximity wins over the strength of the transition dipole of the tag.

Interestingly, there is a rather strong cross peak at 1600/1570. The Tol moiety features a mode computed at ca. 1570 cm^–1^, but it is over 10-fold weaker than that at 1600 cm^–1^. However, the 1600/1570 cross peak is strong at about a half of the diagonal peak at 1600 cm^–1^. The strength of the cross peak originates from a large off-diagonal 1600–1570 anharmonicity, computed at 7.4 cm^–1^, which is much larger than the diagonal anharmonicity of the mode at 1600 cm^–1^, computed at ca. 1 cm^–1^. The pair of diagonal and cross peaks can serve for identifying Tol moieties in 2DIR spectra. The waiting-time trace of this cross peak follows the trace of the 1600 diagonal peak (Figure 5B, blue line) and shows no coherent oscillations.

**Figure 6:**
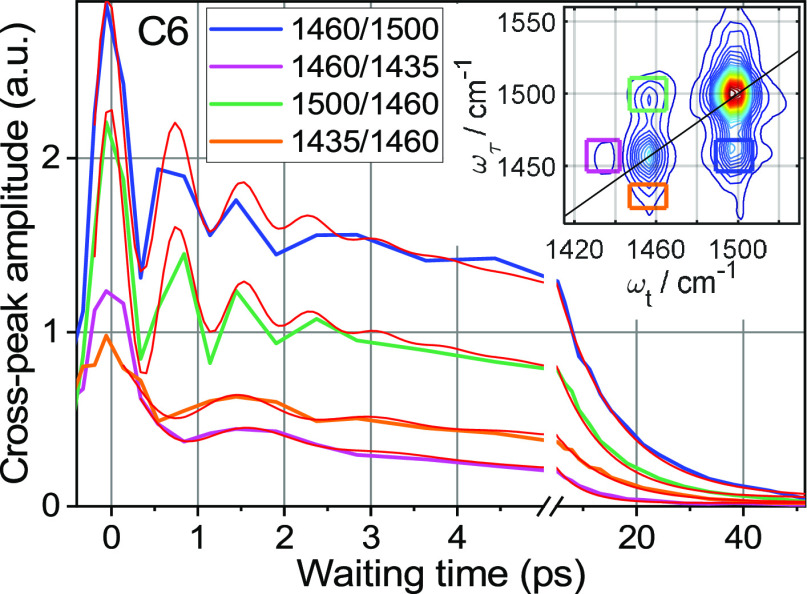
Waiting-time dependence of the indicated cross peaks for C6. The inset shows the 2DIR spectrum measured at 4.3 ps with color-matching boxes indicating the cross-peak integration regions. Thin red lines show fits of the traces with a function *y* = *y*_0_ + *A*_1_ × exp(−*T*/*T*_1_) + *A*_2_ × exp(−*T*/*T*_2_) × cos(2π*T*/*T*_0_ + φ). The fit resulted in the oscillation period, *T*_0_, of 0.78 ± 0.02 ps for both cross peaks involving the 1460 and 1500 cm^–1^ peaks and of 1.6 ± 0.1 ps for the peak at 1435/1460. The oscillation damping time, *T*_2_, was 0.8 ± 0.1 ps for the 1460/1500 and 1500/1460 peaks and 1.2 ± 0.3 ps for the peak at 1435/1460. The overall decay time, *T*_1_, is about 10 ps for all three cross peaks. A complete list of fit parameters is given in Table 5.

Multiple cross peaks were observed in the fingerprint region (see Figure 6 inset), and their waiting-time dependences were analyzed. Only one of these cross peaks shows a clear rise time, but several cross peaks show coherent oscillations as a function of the waiting time. We first discuss the oscillations, then analyze the cross-peak decay times, and then discuss the cross peak showing a rise.

#### Coherent Oscillations in Cross Peaks

The presence of oscillations in a cross peak indicates that a coherent superposition of strongly coupled states is excited by a short mid-IR pulse.^54,71,72^ Therefore, coherent oscillations in 2DIR spectra can help identify modes that belong to the same moiety as their strong coupling requires significant spatial overlap. Several 2DIR cross peaks of C2 and C6 show coherent oscillations. These include the cross peaks between the two strongest peaks, 1500 and 1460 cm^–1^ (1500/1460 and 1460/1500), as well as the cross peaks among the 1460 and 1435 cm^–1^ peaks (1435/1460 and 1460/1435), Figure 6. The period of oscillations, *T*_0_, corresponds to the beating frequency as Δν (in cm^–1^) = 1/(*cT*_0_), where *c* is the speed of light under vacuum. The computed beating frequencies, 43 ± 1 cm^–1^ for 1500/1460 and 1460/1500 and 21 ± 1 cm^–1^ for 1435/1460, are expected to match the energy gap between the involved states excited coherently – the two frequencies of the cross peak. Indeed, the frequency difference between the peaks at 1500 and 1460 cm^–1^ for C6 is 43.2 cm^–1^ (1500.1–1456.9 cm^–1^), which matches well the oscillation frequency. The function used for fitting the data is described in Figure 6 caption, while the fit parameters are given in Table 5.

**Table 5:** Fit Parameters^a^ for Several Cross Peaks for C6

cross peak	*T*_1_, ps	*T*_2_, ps	*T*_0_, ps	φ	*A*_2_/*A*_1_	oscillation freq., cm^–1^	dominant contribution
1460/1500	11.3 ± 0.7	0.79 ± 0.1	0.78 ± 0.02	0.05	0.47	43 ± 1	F_5_Ph/F_5_Ph
1500/1460	9.8 ± 0.8	0.79 ± 0.1	0.78 ± 0.02	0.01	0.70	43 ± 1	F_5_Ph/F_5_Ph
1435/1460	9.8 ± 0.7	1.3 ± 0.4	1.6 ± 0.1	0.07	0.33	21 ± 1	Tri/Tri
1460/1435	5.1 ± 0.3	0.9 ± 0.2	1.7 ± 0.2	–0.05	0.51	20 ± 1	Tri/Tri

a See Figure 6 caption.

Note that the 1500 and 1460 cm^–1^ peaks have dominant contributions from the F_5_Ph ligand. Their cross-peak oscillations are expected to be dominated by coherent excitation of the two F_5_Ph modes, but a smaller contribution from coherent excitation of the modes at Tri is also expected. Because of reduced coupling, it is less likely that the Tol modes will be excited coherently as the group of Tol modes at 1500 cm^–1^ belongs to the Ph ring motion, while those at 1460 cm^–1^ are due to CH_3_ bending, resulting in smaller coupling.

The peak at 1435 cm^–1^ belongs exclusively to Tri. Therefore, the oscillations of the 1435/1460 cross peak have to be due to coherent excitation of the modes at the Tri moiety. The presence of oscillations proves that both modes of the cross peak originate from the same moiety, Tri, permitting to identify a minor Tri contribution within the 1460 cm^–1^ peak, predicted by the DFT-based peak assignment (Table 1). How strong is the contribution to the cross peak of the 1460 cm^–1^ mode of F_5_Ph and the Tri mode of 1435 cm^–1^? Such a cross peak is expected to be weak at waiting times close to zero. Indeed, the amplitude of the oscillations is significant, *A*_2_/*A*_1_ = 0.33, so the cross peak is dominated by the Tri peaks, at least at small waiting times.

### Cross-Peak and Diagonal-Peak Decay Times

#### Slow Decay Components for Diagonal Peaks

While the fast component of a diagonal peak is attributed to the lifetime of the largest peak contributor, the slow decay component of the diagonal peak reflects its cooling time, observed via the mode coupling to low and medium frequency modes (Table 6). Therefore, the slow decay components of diagonal-peak traces for the modes residing at the same moiety should be similar. Indeed, both modes of the Tri moiety, 1435 and 1530 cm^–1^, feature similar cooling times of ca. 7 ps. The cooling times for the diagonal peaks at 1460 and 1500 cm^–1^ are also similar at 10 ± 1 ps, as both peaks reside predominantly at F_5_Ph.

**Table 6:** Exponential Decay Parameters for Cross Peaks for C6

cross peak	*t*_1_, ps	*T*_max_, ps
1500/1435^a^	7.5 ± 0.4	0
1435/1500^a^	15.7 ± 1	7.0
1530/1500	7.6 ± 0.3	0
1500/1530	6.3 ± 0.3	0

a Coherent oscillations observed.

#### Tol-Dominated Peaks

The decay time for the diagonal peak at 1600 cm^–1^, representing Tol moieties, is exceptionally long at 27 ps (Figure 5B, blue). Interestingly, the decay times for two cross peaks with the reporter at 1600 cm^–1^ (1500/1600 and 1460/1600) are also the same at ca. 27 ps (Figure 5B), despite the fact that the strongest contributors to the 1500 and 1460 cm^–1^ peaks reside at F_5_Ph. This similarity indicates that all three cross peaks are dominated by the Tol/Tol type cross-peak contributions. In other words, for the 1500/1600 cross peak the Tol-localized modes are contributing as tags, not the strongest 1500 peak contributor of F_5_Ph. At the same time, the cross-peak contribution associated with the energy transfer between the F_5_Ph and Tol ligands, expected to feature a rise in the waiting time dependence, is fully masked by a much stronger Tol/Tol type cross-peak contribution. Otherwise, either a cross-peak rise would be observed or the decay time would be longer, affected by delayed energy arrival to the reporter site.

#### Tri-Dominated Peaks

**Figure 7:**
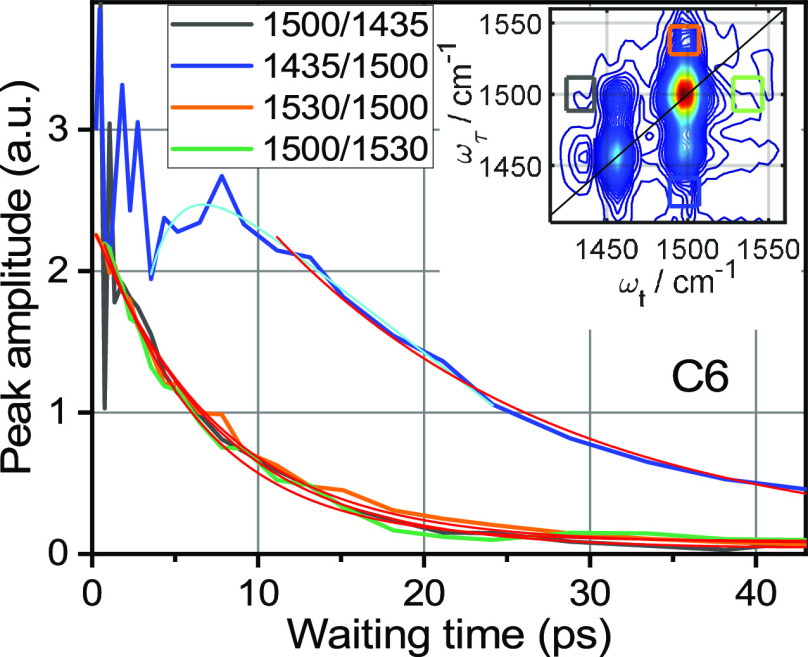
(A) Scaled waiting-time traces for indicated cross peaks for C6. The traces were fitted globally with an exponential decay function (red lines), see results in Table 6. The 1435/1500 cross peak was also fitted with an asymmetric double sigmoidal function (cyan, see Table 5). (B) Exponential decay times measured are summarized for each diagonal and cross peak, also reported in Tables 4–6. The vertical and horizontal lines are color coded to indicate FTIR contributions originated from different ligands, F_5_Ph (red), Tri (blue), and Tol (green).

The diagonal peaks of Tri modes at 1435 and 1530 cm^–1^ feature the same cooling time of ca. 7 ps. The slow decay components are nearly the same for a range of cross peaks involving Tri modes, such as 1500/1435, 1469/1435, 1530/1500, and 1500/1530 (Figure 7A, cyan color). This match suggests that these four cross peaks are dominated by local Tri modes, while the Tri/F_5_Ph and F_5_Ph/Tri type cross peaks, involving energy transfer between the ligands, cannot compete amplitude-wise with the local Tri/Tri type cross peaks.

#### F_5_Ph-Dominated Peaks

The diagonal peaks for the F_5_Ph-residing modes at 1500 and 1460 cm^–1^ show a characteristic cooling time of ca. 10 ps. Their cross peaks, 1500/1460 and 1460/1500, show similar cooling times of 10 ± 1 ps, characteristic of cooling of the F_5_Ph moiety.

#### Cross Peaks at 1435/1500 and 1435/1460

The cross peaks at 1435/1500 and 1435/1460 can have two contributions: one from spatially close Tri/Tri type modes, which may show coherent oscillations, and another of Tri/F_5_Ph type, which is expected to show an amplitude rise with the waiting time. Only one cross peak in the fingerprint region is showing a rise time and a clear maximum, that is, the 1435/1500 peak in C6 (Figure 7A). It shows small amplitude oscillations (∼10%) at early times, indicating a Tri/Tri contribution. The Tri/Tri type cross peak decays with ca. 7 ps, the cooling time of the Tri moiety. Because its contribution diminishes so rapidly, we can clearly see a cross-peak rise associated with the 1435(Tri)/1500(F_5_Ph) cross peak, which peaks at ca. 7–8 ps. This time is slightly shorter than *T*_max_ for the ν_C≡C_/1500 cross peak of 9.7 ps, supporting the picture that excited ν_C≡C_ relaxes into the Tri modes, away from the F_5_Ph ligand, resulting in a longer time. No rise for the 1435/1500 cross peak is found in C2, likely because the rising 1435(Tri)/1500(F_5_Ph) peak contributor is expected to be earlier by 2–3 ps, which is then hidden by the decaying 1435(Tri)/1500(Tri) peak contribution. The 1435/1460 cross peak does not show a resolved maximum, likely due to its small amplitude compared to stronger Tri/Tri contribution as the Tri mode within the 1460 cm^–1^ peak is much brighter than that within the 1500 cm^–1^ peak. The exponential decay times measured for each diagonal and cross peak for C6 are summarized graphically in Figure S12.

Detailed assessment of the cooling times of different ligands (moieties) permits understanding the origin of the cross peaks and revealing different contributions to cross peaks with multiple overlapping modes. A significant difference in the effective cooling times for the modes at Tri, F_5_Ph, and Tol moieties was found; the cooling times are at ca. 7, 10, and 27 ps, respectively. To further understand the vibrational energy transport within these complex molecules, we computed relaxation pathways of various excited modes.

### Computing Vibrational Relaxation Pathways

**Figure 8:**
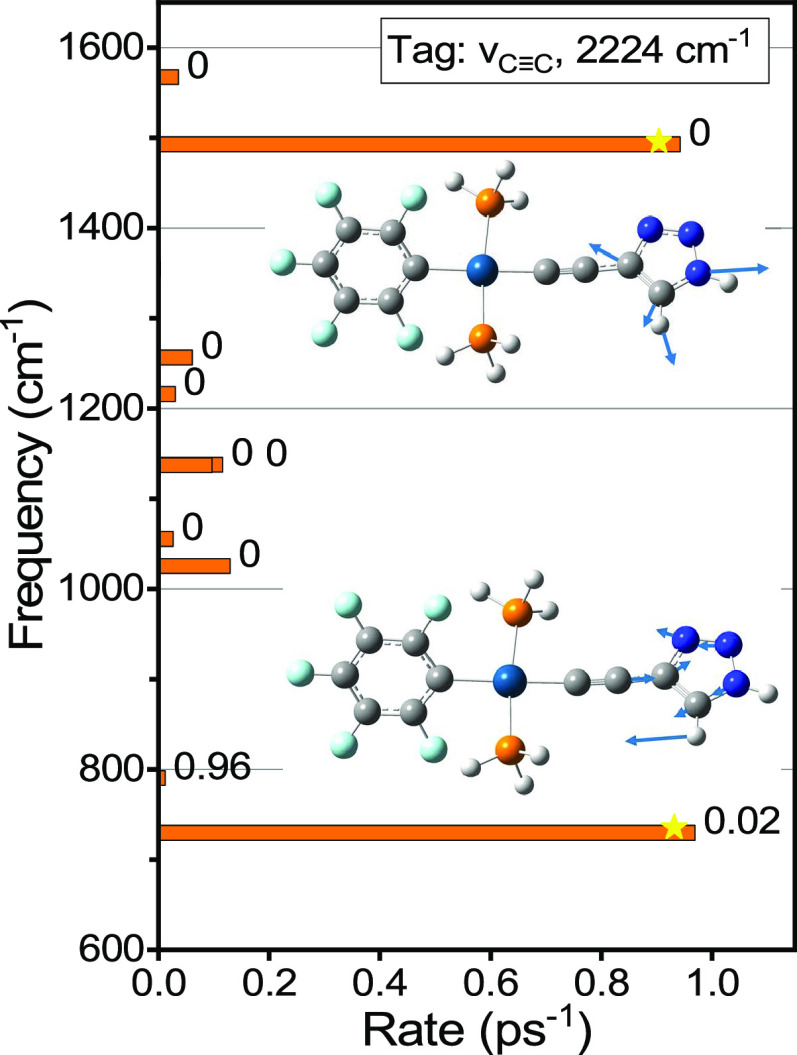
Rates of dominant relaxation channels of ν_C≡C_ computed for C2F (see Figure S7 for C6F). The displacements of the strongly contributing normal modes (labeled with stars) are shown as insets. Delocalization factors, χ, are shown for each normal mode to the right of its rate bar. Note that χ(ν_C≡C_) < 10^–4^.

The whole molecules were too large to perform anharmonic DFT calculations, so such computations were performed on the fragments of the compounds, where the Tol and CH_2_-C_6_H_5_ groups were replaced by hydrogen atoms (labeled as C2F and C6F), as shown in Figures 8 and S7 insets.

Figure 8 shows the dominant relaxation channels for the ν_C≡C_ tag in C2F, presented as rate bars populating various daughter states. Each relaxation daughter mode is labeled with a χ value, (Figure 8, right of bars) which represents the level of mode delocalization between the F_5_Ph and C_*n*_-Tri ligands across the Pt center (excluding motions at the PH_3_ ligands). The modes with χ ∼ 1 reside at the F_5_Ph ligand, while those with χ ∼ 0 reside at the C_2_-Tri ligand. Clearly, the ν_C≡C_ relaxion daughter modes are predominantly (99.5%) residing at the Tri ligand with χ of 0–0.02. The two daughter modes populated the most are at 1492 and 729 cm^–1^ (Figure 8, bars marked with stars). The ν_C≡C_ relaxation pathways in the C6 compound are richer but similarly populate predominantly the modes of the Tri ligand with χ < 0.01 (see Figure S7). Both compounds show negligible relaxation of the ν_C≡C_ tag directly into the F_5_Ph localized modes.

**Figure 9:**
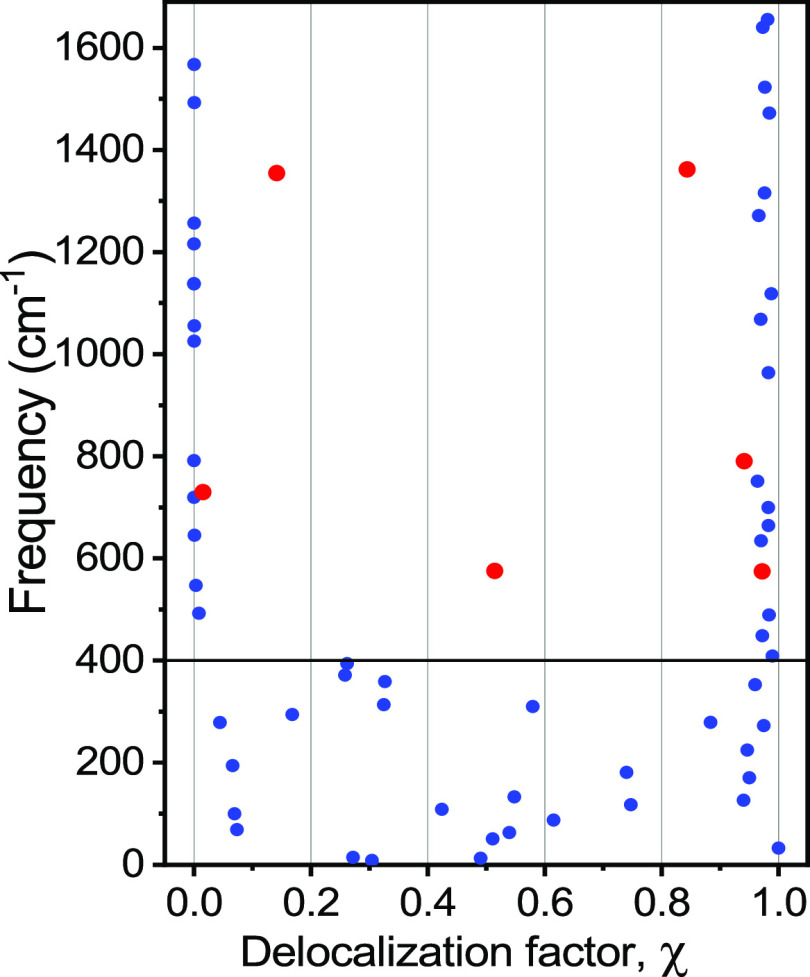
Delocalization factor, χ, for all normal modes below 1650 cm^–1^ in C2F (see Figure S8 for C6F). Three pairs of significantly delocalized high-frequency modes are shown with red circles. Most high-frequency modes (>400 cm^–1^) are localized at either F_5_Ph (χ ∼ 1) or C_2_-Tri (χ ∼ 0) ligands. Low-frequency modes (<400 cm^–1^) are mostly delocalized across the Pt center.

To illustrate the waiting-time dependence on the chain length, we performed a modeling in which the ν_C≡C_/F_5_Ph cross-peak amplitude for C2 and C6 was plotted as a function of the waiting time (Figure 13A and 14A). Reasonable shapes of the waiting-time dependences were obtained with the *T*_max_ values showing similar trends to the experimental values (7.4 ps in C2 and 9.7 ps in C6). Detailed analysis showed the presence of a very large number of relaxation pathways in the energy relaxation process, *vide infra*. To identify the pathways leading to energy crossing the Pt center, a detailed analysis of the mode couplings and delocalization was performed (Figures 9 and S8).

**Figure 10:**
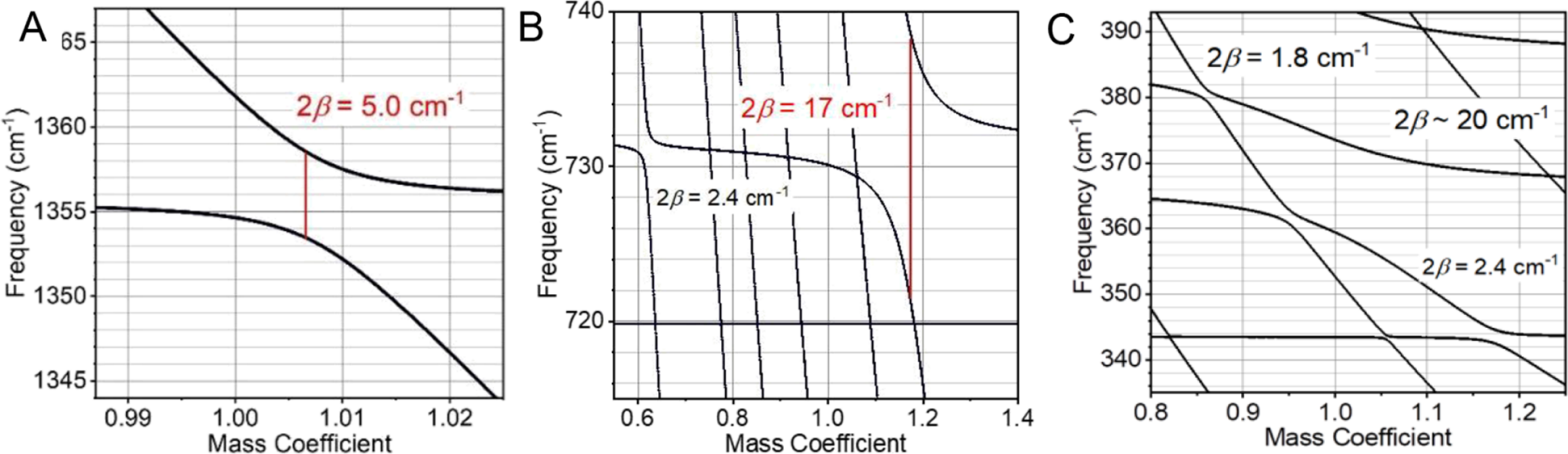
Mode frequency of delocalized pairs (A) 1354 and 1362 cm^–1^, (B) 729 and 790 cm^–1^, and (C) frequencies around 360 cm^–1^ as a function of the mass scaling factor for the C and F atoms of the F_5_Ph moiety. Vertical red lines show the frequency jump (2β) of the observed modes.

We found that despite some similarities of bond types in the F_5_Ph or C_2_-Tri ligands, most of their high-frequency modes are localized on either of the ligands. However, there are a few pairs of normal modes showing significant delocalization. To identify potential delocalization, the coupling strength among the local modes at F_5_Ph and C_2_-Tri were computed by varying the masses of the 11 atoms of F_5_Ph in small increments and computing normal modes for each mass value using the Hessian matrix obtained via DFT normal-mode analysis. As a result of the mass change of the F_5_Ph moiety, an avoided splitting is observed for the interacting modes which equals 2β, where β is the interaction energy (Figure 10). We found that the coupling of local modes across the Pt center does not exceed ∼20 cm^–1^, while typical couplings are less than 2 cm^–1^. Only two pairs of high-frequency modes (>400 cm^–1^) were found to be coupled strongly enough to result in mode delocalization at the actual atomic masses of F_5_Ph (χ = 1). One pair involves partially delocalized modes at 1355 cm^–1^ (χ = 0.14) and 1362 cm^–1^ (χ = 0.84) with 2β = 5 cm^–1^ (labeled with red circles in Figure 9). Another pair involves modes at 730 cm^–1^ (χ = 0.015) and 790 cm^–1^ (χ = 0.94) with 2β = 17 cm^–1^ (Figure 10B). Relatively small couplings require rather precise match of the site frequencies to result in delocalization, limiting the number of delocalized modes. Interestingly, the low-frequency modes (<400 cm^–1^) are predominantly delocalized across the Pt center (Figure 9).

#### Origin of Mode Coupling across the Pt Center

**Figure 11:**
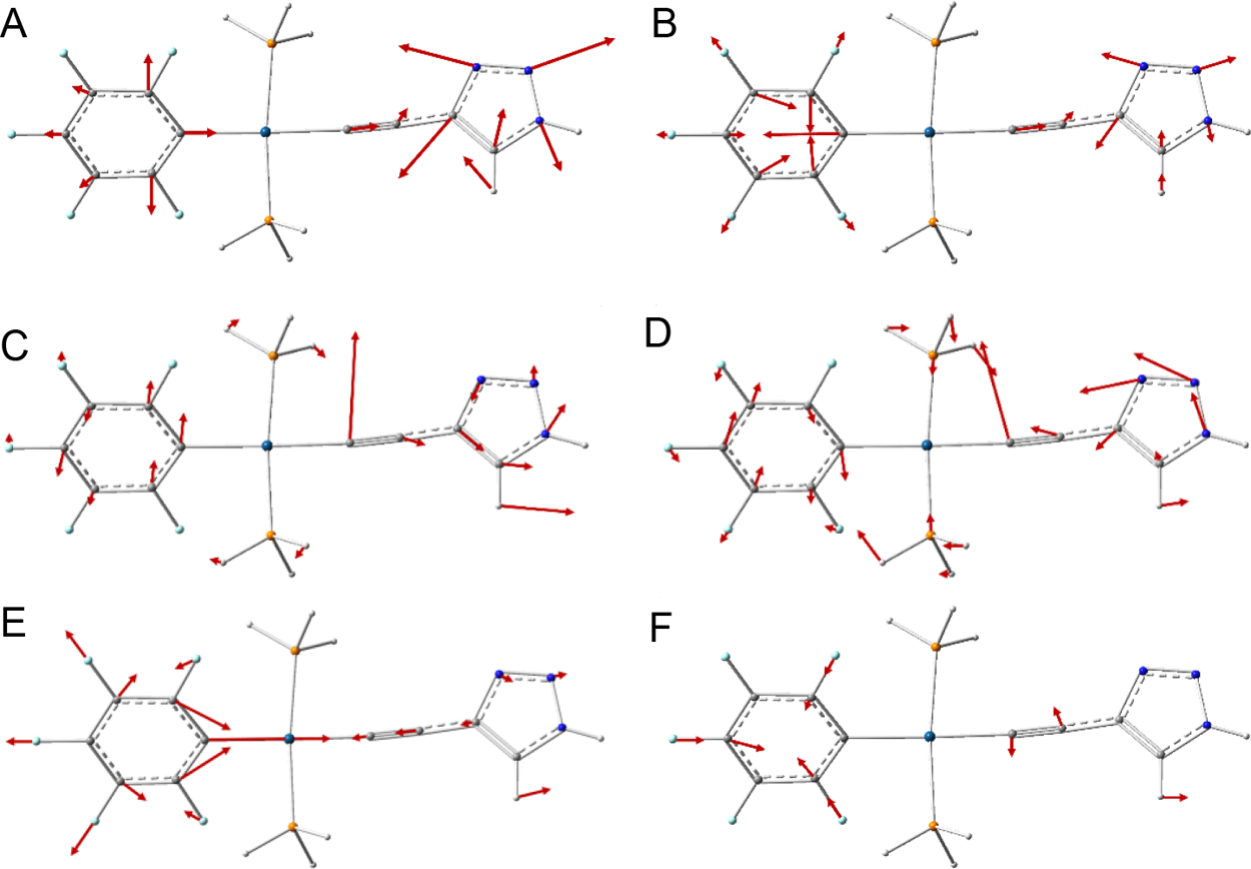
Displacements for delocalized normal modes at (A) 1355 cm^–1^, (B) 1362 cm^–1^, (C) 394 cm^–1^, (D) 359 cm^–1^, (E) 790 cm^–1^, and (F) 575.5 cm^–1^.

Two types of coupling mechanisms are possible: through-space electrical coupling and through-bond mechanical coupling. The modes of the two pairs feature IR intensities not exceeding 60 km/mol and the effective distances well exceeding 4 Å (carbon–carbon distance across Pt is ca. 4.1 Å). For such weak modes and distances over 5.3 Å, the electrical dipole–dipole coupling is computed to be smaller than 0.25 cm^–1^ (see the SI). We found that the modes with the largest coupling strength (2β > 5.0 cm^–1^) are coupled mechanically, involving a change of the C–Pt–C distances, as for modes of a coupled pair at 1350 and 1362 cm^–1^, Figure 11A,B. While the Pt atom is heavy and does not move much, both adjacent carbon atoms move in the Pt–C stretching fashion as in-phase and out-of-phase combinations for the modes of the pair. The energy gap of the local modes is larger than the coupling leading to only a partial mixing of the site states. The energy match is somewhat accidental, as the two ligands are different. At the same time, both ligands feature similar bond types, carbon–carbon with a bond order of 1.5, which facilitates the energy match and mixing of the local modes featuring Pt–C stretching motions. Another type of local motion that leads to strong coupling between the local modes of the two ligands involves C–Pt–C angle change (Figure 11C,D). Such motions are present in many local modes with 250–400 cm^–1^ frequencies, ensuring delocalization of normal modes in this frequency region (Figure 9). The lower-frequency modes, <250 cm^–1^, are delocalized over the whole compound, as expected.

**Figure 12:**
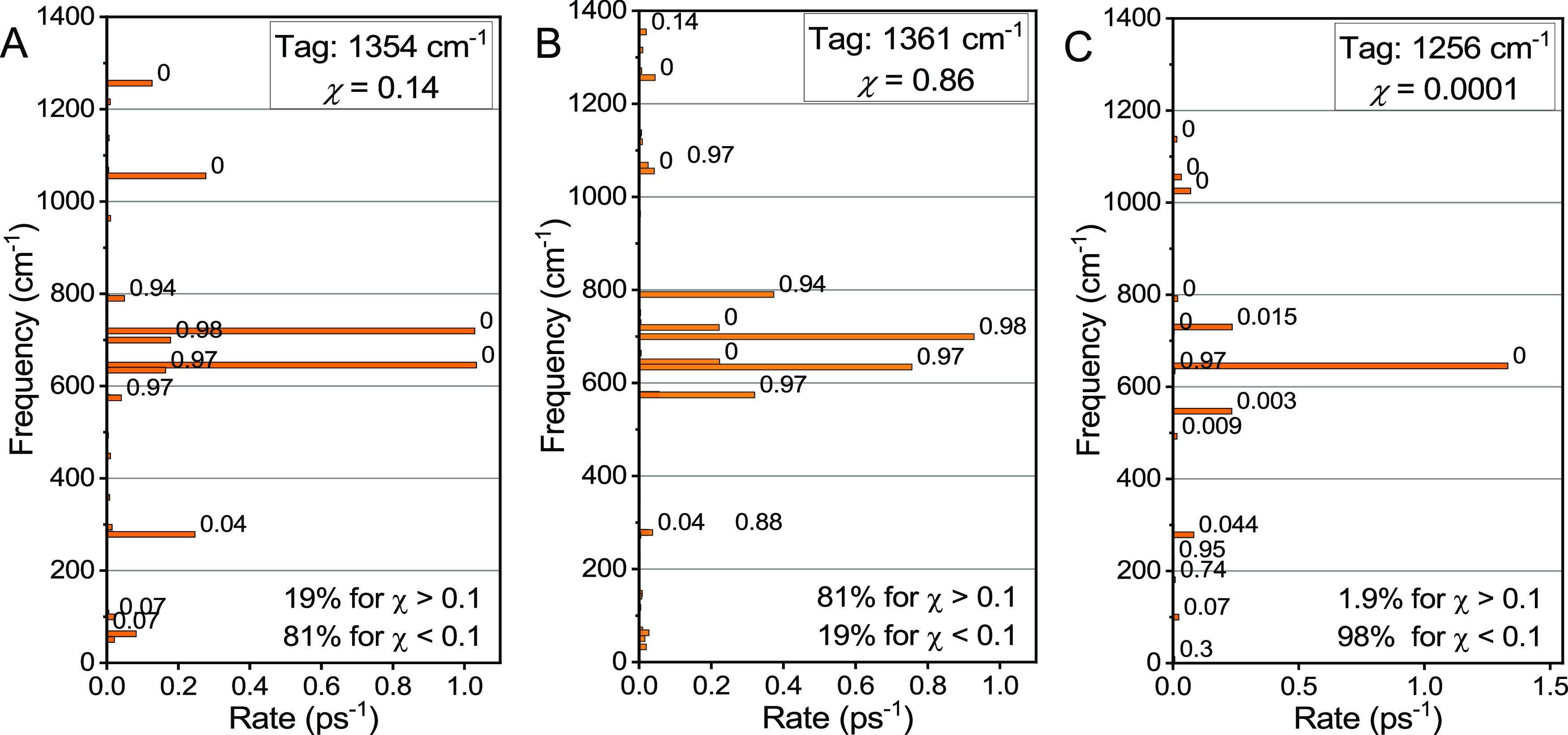
Rates of dominant relaxation channels in C2F for two delocalized modes (A) 1361 cm^–1^ and (B) 1354 cm^–1^ and one mode localized at the C_2_-Tri ligand, (C) 1256 cm^–1^. The values on the right of each bar represent the mode delocalization factor, χ.

Vibrational relaxation is governed by third-order force constants; to be significant they require spatial overlap of the parent and daughter modes. Figure 12 illustrates this statement presenting relaxation pathways of the three modes, two modes of the partially delocalized pair at 1361 and 1354 cm^–1^ with a delocalization extent of 0.86 and 0.14, respectively, and a high-frequency mode at 1256 cm^–1^ located at the C_2_-Tri ligand (χ = 0.001). The mode at 1354 cm^–1^ (χ = 0.14) relaxes predominantly to modes within C_2_-Tri (81%) but also relaxes to modes localized at the F_5_Ph ligand (19%), thus providing a high-frequency (600–800 cm^–1^) pathway to F_5_Ph across the Pt center (Figure 12A). The relaxation pathways of the 1361 cm^–1^ mode (χ = 0.86) are complementary to those for 1354 cm^–1^; 19 rate percent of the pathways involve energy passage from F_5_Ph to C_2_-Tri (Figure 12A,B insets).

The daughters of the 1256 cm^–1^ mode (χ = 0.001) relaxation located predominantly (>98 rate percent) at the same ligand as the parent mode (Figure 12C). Therefore, to have sizable relaxation rates across the Pt center, the parent mode should be delocalized significantly across the center. If such a delocalized parent mode became excited, it relaxes into modes on both sides of the Pt center. At the same time if a mode is localized at C_*n*_-Tri, it predominantly relaxes into the modes at C_*n*_-Tri, resulting in negligible energy transfer across the Pt center (Figure 12C). As the number of delocalized high-frequency modes is small, there are a small number of energy transfer pathways across the Pt center.

Most vibrational modes lower than 400 cm^–1^ are delocalized (Figure 9). A strong coupling of local modes of F_5_Ph and C_2_-Tri in the 250–400 cm^–1^ region originates from local modes resulting in a change of the C–Pt–C angle (bending). The low-frequency modes (<250 cm^–1^) naturally involve motion of the whole molecule. The delocalized modes (<400 cm^–1^) feature a significant coupling to high-frequency modes on each side of the Pt center. If excited, they perturb the reporter at the F_5_Ph moiety causing an increase of the C≡C/reporter cross peak amplitude. It is important to note that excitation of delocalized low-frequency modes does not lead to energy transfer across the Pt center, as the energy excess remains on both sides of the Pt center.

**Figure 13:**
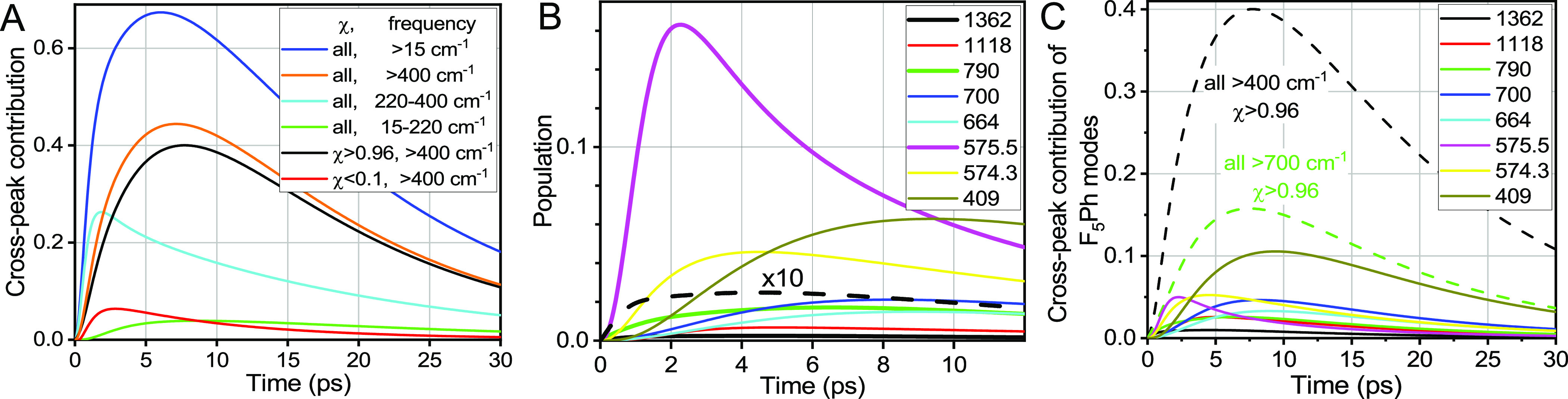
(A) Contributions of different groups of modes to the ν_C≡C_/1500(F_5_Ph) cross peak computed for C2F. The groups are formed based on the frequency (>15, >400, 220–400, or 15–220 cm^–1^) and delocalization factor, χ (all, >0.96, or < 0.1). (B) Waiting-time population traces for high-frequency modes of F_5_Ph (χ > 0.96) and the mode at 575.5 cm^–1^ (χ = 0.51). 10-fold normalized population trace for 1362 mode is also shown by a black dashed line. (C) Cross-peak contributions of the modes shown in panel B. The overall contributions of all F_5_Ph modes (χ > 0.96) of frequencies >700 cm^–1^ (green) and >400 cm^–1^ (black) are shown with dashed lines.

The relaxation dynamics initiated by relaxation of the excited ν_C≡C_ mode was computed, and the excess populations of every mode (Figure 13B) and their contributions to the ν_C≡C_/1500(F_5_Ph) cross-peak were extracted and analyzed (Figure 13A,C). The IVR process of ν_C≡C_ populates every mode in the compound but to a different extent and at different times. The maximum computed for all modes higher than 15 cm^–1^ is peaking at ∼6.1 ps (Figure 13A, blue line). To understand which modes are involved in transferring energy from the C_*n*_-Tri ligand to F_5_Ph, we analyzed contributions from different groups of modes to the cross-peak (Figure 13A).

As expected, we found that the ν_C≡C_/1500(F_5_Ph) cross-peak is dominated by contributions of the modes at F_5_Ph (those with high χ, Figure 13A, black) as they are well coupled to the reporter located at F_5_Ph. The high-frequency modes, >400 cm^–1^, are mostly localized at either ligand so they are easily sorted into the modes at F_5_Ph (large χ, black line) or C_2_-Tri (small χ, red line). The modes with χ > 0.96 contribute the most (black line) with a peak at 7.7 ps. High-frequency modes with χ < 0.1 (red line) contribute much less, peaking at ca. 3 ps.

Many modes in the 220–400 cm^–1^ window are delocalized over the two ligands due to the motion involving the C–Pt–C angle change (Figure 11C,D); their contribution is shown in Figure 13A, cyan. Interestingly, the contribution is significant but peaking at earlier delay times of ∼2 ps, thus shortening the *T*_max_ values for the ν_C≡C_/F_5_Ph cross peaks. Low-frequency modes, <220 cm^–1^ (Figure 13A, green), are not contributing much to the cross peak, mostly because of their small coupling to the high-frequency reporter at F_5_Ph.

Based on the match of the experimental *T*_max_ values for the ν_C≡C_/1500(F_5_Ph) cross peak (7.4 ps) and the computed *T*_max_ (Figure 13A), we conclude that the high-frequency modes at F_5_Ph (high χ) determine the *T*_max_ values.

To understand the pathways populating the high-frequency modes at F_5_Ph, we analyzed the population dynamics of these modes. Figure 13B shows the high-frequency modes with χ > 0.5 having the highest populations. Importantly, the modes of F_5_Ph that are populated the earliest and to the largest extent contribute the most to the energy transfer process from the C_2_-Tri ligand to F_5_Ph. We found that such modes involve those modes of the coupled pairs: 575.5, 790, and 1362 cm^–1^ (Figure 13B). The populations for the 790 and 1362 cm^–1^ modes are rising with no delay, indicating that they are daughter modes of the ν_C≡C_ relaxation. However, their contributions are not very large, even though they carry larger energy. The mode at 575 cm^–1^ (Figure 13B, magenta) belongs to a coupled pair involving mixing of the CCC bending at C_2_-Tri and F_5_Ph deformation motions (Figure 11). It is not populated directly from ν_C≡C_, as apparent from the presence of the induction period but is still populated very rapidly leading to a maximum at ∼2.3 ps. Another mode of the pair, 574.3 cm^–1^ (χ = 0.972, Figure 13B, yellow), follows the trace of the 575.5 mode with some lag, peaking at ∼4.5 ps. Figure 13C shows the largest high-frequency contributors to the cross peak. Note that because of a strong coupling among the modes of F_5_Ph, the equilibration among them occurs within a few IVR steps (3–5 ps), so that many F_5_Ph modes became populated after 3 ps. The large contribution of the mode at 409 cm^–1^ (χ = 0.9895) reflects its lower frequency. Nevertheless, the overall contribution of the high-frequency modes (>700 cm^–1^) is very significant (Figure 13C, green dashed line).

To summarize, the high-frequency modes of the mode pairs coupled across the Pt center provide pathways to transfer energy between the two ligands. The high-frequency modes, >400 cm^–1^, are responsible for 66% of the cross-peak amplitude. The modes in the 220–400 cm^–1^ region also contribute significantly, reaching ca. 40% in amplitude, but interestingly, the maximum is reached much earlier than *T*_max_ at 1.9 ps (Figure 13A, cyan). The modes in this range are largely delocalized over the two ligands. This delocalization is caused by the similarity of the groups on each side of the Pt center resulting in similar frequencies of the Pt–C–C bending motion on each ligand. Therefore, the process of energy transfer between the ligands relies on the similarity of the groups at the two ligands in the vicinity of the Pt center and frequencies of their modes.

**Figure 14:**
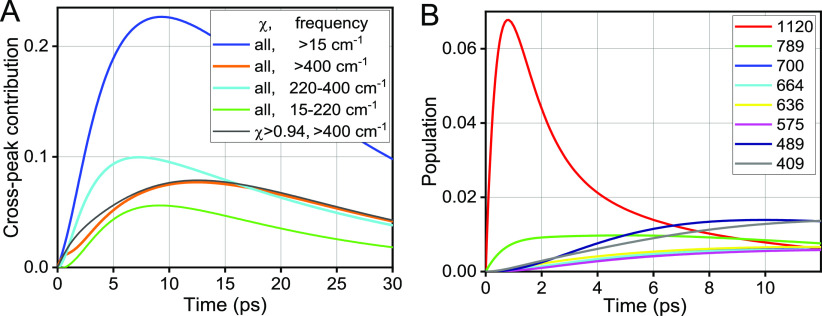
(A) Contributions of different groups of modes to the ν_C≡C_/1500(F_5_Ph) cross peak computed for C6F. The groups are formed based on the frequency (>15, >400, 220–400, or 15–220 cm^–1^) and delocalization factor, χ (all or > 0.94). (B) Waiting-time population traces for high-frequency modes of F_5_Ph (χ > 0.94) contributing the most to the cross peak.

The relaxation pathways of ν_C≡C_ computed for C6F revealed a similar behavior to that for C2F: only high-frequency modes of strongly coupled pairs showed a significant energy transfer from C_6_-Tri to F_5_Ph (Figure 14B); the populations of those modes and all high-frequency modes of F_5_Ph, however, reach much smaller values compared to those in C2F (Figure 13B). As a result, the overall relative contribution of energy transfer pathways involving high-frequency modes in C6F (∼35%, Figure 14A) is half of that for C2F. Low-frequency modes, 15–220 cm^–1^ (green) and especially 220–400 cm^–1^ (cyan), show a much larger relative contribution to the cross peak in C6F. Spatial proximity of the centers of the two ligands dictates the outcome of the energy transport process, showing a similar connectivity observed previously for modes within a covalent network.^73,74^

Detailed analysis of relaxation pathways of high-frequency modes enables us to identify specific modes involved in energy transfer across the molecule in C2F and C6F. The efficiency of the energy transfer across the Pt center was found to correlate with the delocalization extent of the parent mode across the center. A negligible energy transport efficiency from the C_*n*_-Tri to F_5_Ph moieties was found for the modes with the extent of delocalization at the F_5_Ph moiety smaller than 1%. The overall cross-peak waiting-time dynamics in C2F is dominated by the energy transfer via high-frequency modes (>400 cm^–1^), while low-frequency modes dominate for C6F.

The energy transfer time, *T*_max_, appears to be different when the tag and reporter are reversed (Tables 2, 3), indicating different contributions of high- and low-frequency energy transfer channels in the two cases. The transport channels associated with high-frequency modes are expected to alter with a change of the tag as the relaxation channels are tag-specific and will not involve the same modes. Such changes are found for C2, *T*_max_(ν_C≡C_/1500) = 7.4 ps and *T*_max_(1500/ν_C≡C_) = 9.7 ps, indicating the importance of the high-frequency mode transport initiated more efficiently by ν_C≡C_ compared to the 1500 cm^–1^ mode initiation. A similar effect is observed for the ν_C≡C_/1460 and 1460/ν_C≡C_ peaks, although the Tri contribution to the peak at 1460 cm^–1^ makes quantitative assessment difficult. On the other hand, the similarity of the transport times for reversed tag and reporters likely points at a dominant contribution of the low-frequency modes in the thermalization process, as, for example, for the ν_C≡C_/1500 and 1500 /ν_C≡C_ cross peaks for C6 (9.7 and 11 ps, respectively). Note that no strongly coupled high-frequency local modes of Tol and C_*n*_-Tri or Tol and F_5_Ph were found. Therefore, we concluded that the thermalization to and from Tol moieties occurs via low-frequency modes.

## Conclusions

Rather large compounds, C2 and C6, were investigated with 2DIR and RA 2DIR spectroscopy methods, and waiting-time dependences of a large number of cross and diagonal peaks were measured. Comprehensive analysis of their waiting-time traces enabled (i) better assignment of peaks in the FTIR spectrum to different functional groups, (ii) finding delineating kinetic parameters for several functional groups, which include coherent oscillations of specific cross peaks, energy transfer and cooling parameters, and (iii) understanding the mechanism of energy transfer between the ligands.

We deciphered characteristic 2DIR features of several functional groups, which can help identifying these groups using 2DIR spectroscopy. The mode lifetimes and cooling times of several high-frequency modes at F_5_Ph, C_*n*_-Tri, and Tol were found to be characteristic of each group. The Tol groups in phosphine ligands are found well isolated from the rest of the compounds showing very slow energy dissipation away from the Tol moieties. The Tol diagonal peak at 1600 cm^–1^ and cross peaks involving Tol modes show a two-exponential decay with the fast component of ca. 1 ps, corresponding to the excited mode lifetime, and exceptionally slow component of 26 ps corresponding to energy dissipation from the Tol moieties to other parts of the complex and to the solvent. The amplitude of the slow component is exceptionally high, almost equal to the amplitude of the fast component. It is likely that the excess energy can migrate between different Tol moieties of the same phosphine ligand, but the rate of this process is not known. Note that such energy migration does not change the slow component of the diagonal- and cross-peak kinetics as the excess energy does not leave the Tol moieties. Another interesting feature of the Tol spectra is the presence of a rather strong cross peak at 1600/1570. The anharmonic coupling of the two modes is ca. 7-fold larger than the diagonal anharmonicity of the 1600 cm^–1^ mode, resulting in comparable intensities of the 1600/1570 cross and 1600 cm^–1^ diagonal peaks. This pair of peaks can serve to identifying the Tol motifs in 2DIR spectra of more complex compounds.

We found that coherent oscillations are useful to identify contributions of coupled modes to an IR peak involving multiple overlapping components. One of the advantages of 2DIR spectroscopy, established at the dawn of the field,^75^ is the ability of 2DIR cross peaks to identify modes under a complicated spectrum via their anharmonic coupling. Observation of coherent oscillations enables making a further step and identifying pairs of strongly coupled modes even if both are hidden under a complicated linear spectrum of the compound. Coherent oscillations in a cross peak indicate that the two modes are strongly harmonically coupled and that their coherent superposition was excited by the IR pulses. Strongly harmonically coupled modes can be found in a variety of moieties, including various ring structures, Ph, Tol, F_5_Ph, imidazole, thiophene, cyclohexane, and oligomeric motifs. As a result, such structural motifs can be identified in 2DIR via coherent oscillations of the cross peaks.

DFT calculations, IVR modeling, and evaluation of mode couplings were used to understand the details of the energy transfer and relaxation pathways. Because of closeness of the alkyne bridge to the F_5_Ph ligand and similarity of some bond types in them, a rather efficient energy transfer from ν_C≡C_ to modes at F_5_Ph was found. The requirements for energy transport across the Pt center at high-frequency modes have been formulated and illustrated. They involve coupling and delocalization of the local modes at two sides of the metal center. The transport from ν_C≡C_ to F_5_Ph in C2 occurs predominantly via three mode pairs delocalized over both ligands, featuring frequencies of 1362/1354, 730/790, and 575.5/574.3 cm^–1^ and involving C–Pt–C stretching (first two pairs) and bending (last pair) motions (Figure 11). These motions on each ligand are coupled across the Pt center with a coupling strength of 5–20 cm^–1^, which is sufficient to cause significant delocalization and to provide efficient energy relaxation pathways between the ligands. The similarity of the bond types at the F_5_Ph and C_*n*_-Tri ligand helps the frequency match of their local states, thus causing delocalization. However, an accidental frequency match for local states across the Pt center, supported by their significant coupling strength, can lead to delocalization in the same way.

The modes associated with C–Pt–C bending motion (250–400 cm^–1^) are also involved in energy transfer between the ligands, but their contribution is smaller than that of the high-frequency modes in C2. Low-frequency modes (<250 cm^–1^) are largely delocalized across the Pt center in C2 and C6. Population of such modes occurs via IVR as vibrational relaxation proceeds, reaching maximum at ca. 7–8 ps. When excited, such modes affect the reporter frequency wherever it is located in the molecule. The IVR process involving the modes delocalized over the whole compound does not lead to energy migration (transport).

As typical lifetime of a high-frequency mode in compounds with more than 10 atoms is 1–2 ps and a few IVR steps lead to dominant population of low-frequency modes, the time window for energy transport in medium-size compounds, such as C2 and C6, is limited to ca. 5 ps. The observed *T*_max_ values for the ν_C≡C_/(modes at Tol) greatly exceed this limit; thus, low-frequency modes determine the *T*_max_ values for such cross-peak types. Note that for the ν_C≡C_/F_5_Ph cross peaks, the low-frequency modes in the 250–400 cm^–1^ region contribute significantly, while not for the ν_C≡C_/Tol cross peaks, where the delocalized modes <250 cm^–1^ are contributing the most to the growth of the cross-peak amplitude. A positive correlation between the tag-reporter distance and the *T*_max_ time is observed as expected, emphasizing the value of the RA 2DIR method for mode assignment. The distance correlation is clear in comparing the *T*_max_ values for C2 and C6 (Figures 3 and 4, Tables 2 and 3).

## Supporting Information Available

FTIR spectral modeling for acetylide stretching peaks, normal-mode displacements, additional 2DIR spectra and diagonal and cross peak waiting-time traces; additional computational results include relaxation channels of ν_C≡C_ for C6F, mode delocalization in C6, and electrical dipole–dipole coupling across the Pt center (PDF)
