# Supplementary material for: Tracking Energy Transfer across a Platinum Center
Source: J Phys Chem A. 2022 Jul 26;126(30):4915–30. doi: 10.1021/acs.jpca.2c02017 (PMC9358659; doi:10.1021/acs.jpca.2c02017)
Supplement: Supplementary file 1 — jp2c02017_si_001.pdf [file jp2c02017_si_001.pdf]

Supporting Information

Tracking Energy Transfer Across a Platinum Center

Tammy X. Leong[a], Brenna K. Collins[b], Sourajit Dey Baksi[b], Robert T. Mackin[a], Artem Sribnyi[a], Alexander L. Burin[a], John A. Gladysz[b] and Igor V. Rubtsov[a]*

[a] *Department of Chemistry, Tulane University, New Orleans, Louisiana 70118, United States*

[b] *Department of Chemistry, Texas A&M University, College Station, Texas 77842, United States*

*Corresponding author: irubtsov@tulane.edu

Table of Contents

1. Acetylide Modes
2. Normal Mode Displacement of Toluyl Modes
3. 2DIR Spectra of C2 and C6
4. Percent Contribution of Functional Groups to Normal Modes
5. Diagonal and Cross Peaks in the Fingerprint Region
6. Relaxation Channels of $\nu_{C\equiv C}$ for C6F
7. Mode Delocalization in C6F
8. Electrical Dipole-Dipole Coupling Across Pt Center
9. Comparison the Diagonal Peak Dynamics for C2 and C6

1. Acetylide Modes

The frequencies of the $\nu_{C\equiv C}$ modes were computed via DFT normal-mode analysis. The $\nu_{C\equiv C}$ frequency for C2 was computed at 2217.4 cm^{-1} (75.5 km/mol IR intensity), while observed at 2130 cm^{-1}. The frequency correction factor of 0.961 is required to correct the computed frequency to match the experiment. The $\nu_{C\equiv C}$ frequencies for C6 were computed at 2282.9 cm^{-1} (91.3 km/mol), 2243.7 cm^{-1} (609.7 km/mol), and 2119.0 cm^{-1} (187.2 km/mol). Figure S1 shows the FTIR spectrum of C6 (cyan line), and the line spectrum (red line) frequency corrected with a 0.9543 factor to match the strongest $\nu_{C\equiv C}$ peak. The broadened line spectrum with a Lorentzian fwhm linewidth of 14.5 cm^{-1} is shown with thick green line. The match of the computed spectrum and the experimental FTIR spectrum is not ideal but reasonable; the match can likely be improved by incorporating anharmonic corrections. The line spectrum for C2 is shown with green line and the broadened spectrum with a Lorentzian fwhm linewidth of 20.0 cm^{-1} is shown with thick magenta line. Note that the computed spectrum for C6 was amplitude scaled to get the best match for the strongest peak; the computed spectrum for C2 was scaled with the same factor as for C6. As a result, the computed peak for C2 is ca. 20% smaller than the FTIR peak.

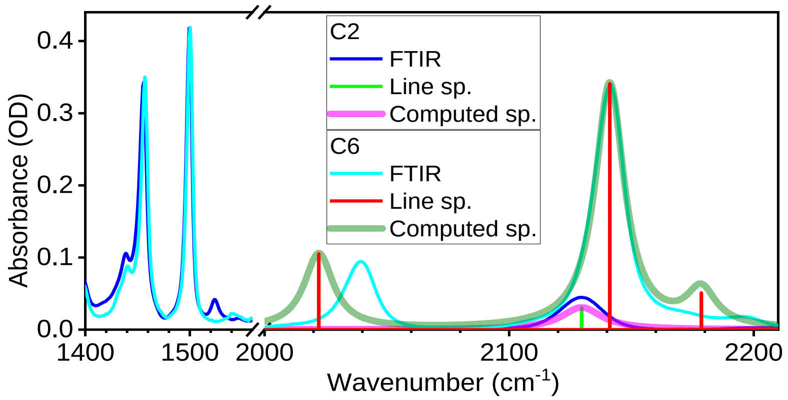

Fig. S1. Experimental FTIR, DFT-computed line, and computed (Lorentz broadened) spectra for C2 and C6.

Table S1. Experimental absorption peaks of $\nu_{C\equiv C}$ for C2 and C6 and their DFT-computed normal mode frequencies.

	Experimental peak, cm^{-1}	Computed peak, cm^{-1}	IR Intensity, km/mol
C2	2130	2217	75
C6	2040	2119	187
	2140	2243	609
	2170	2282	91

Frequency correction factors were 0.961 for C2 and 0.9543 for C6.

Diagonal $\nu_{C\equiv C}$ peaks were measured for C2 (Fig. S2A) and C6 (Fig. S2A). The decay trace of the 2125 cm^{-1} diagonal peak for C2 displayed a fast component of 0.85 ± 0.1 ps (90%) and a slow component of 9.4 ± 0.7 ps (10%). The decay trace of the 2140 cm^{-1} diagonal for C6 displayed a fast component of 1.9 ± 0.1 ps (81%) and a slow component of 11.3 ± 1.3 ps (19%). Note that the decay trace of the 2040 cm^{-1} diagonal peak for C6 has similar waiting time dynamics. The DFT computed lifetimes matched well the experiment with the lifetimes of 0.83 and 1.74 ps for C2 and C6, respectively (see Figs. 8 and S7).

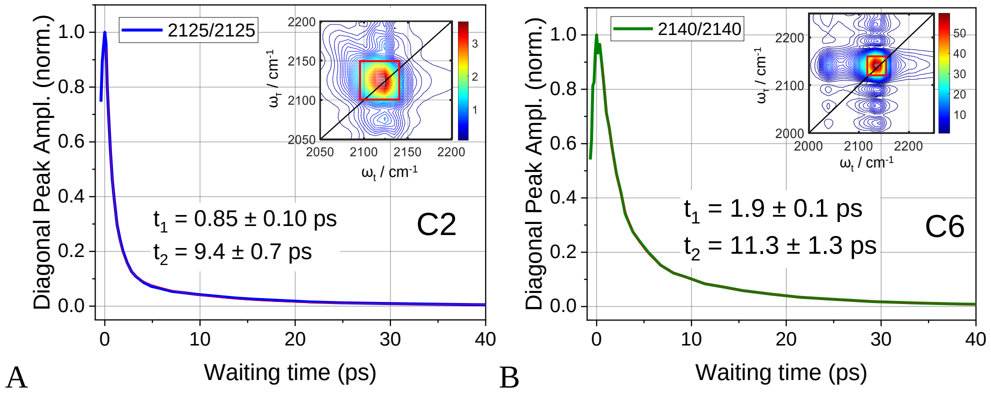

Fig. S2. Scaled waiting-time traces of $\nu_{C\equiv C}$ diagonal peaks for **A.** C2 and **B.** C6. The peaks were fitted with a double-exponential decay function (red lines) The insets in A and B show 2DIR

spectra measured at 2.4 ps and 2.6 ps, respectively, with red boxes indicating the integration regions.

2. Normal Mode Displacement of Toluyl Modes

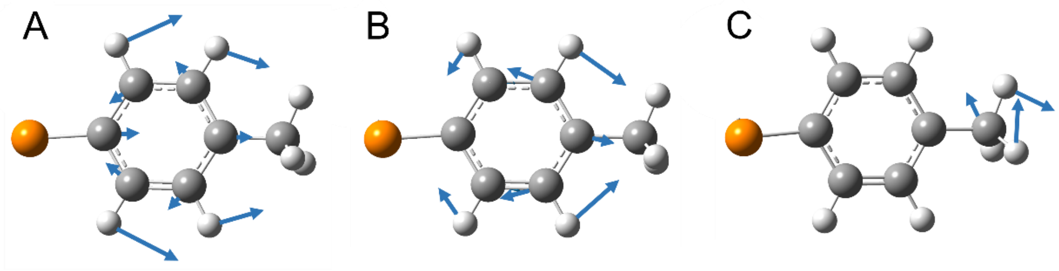

Figure S3. Normal mode displacements of the p-tolyl group modes at about (A) 1500 cm^{-1}, (B) 1600 cm^{-1} and (C) 1460 cm^{-1} (CH_3 asymmetric bending).

3. 2DIR Spectra of C2 and C6

2DIR spectra of C2 and C6 measured under the same IR frequency and scan conditions.

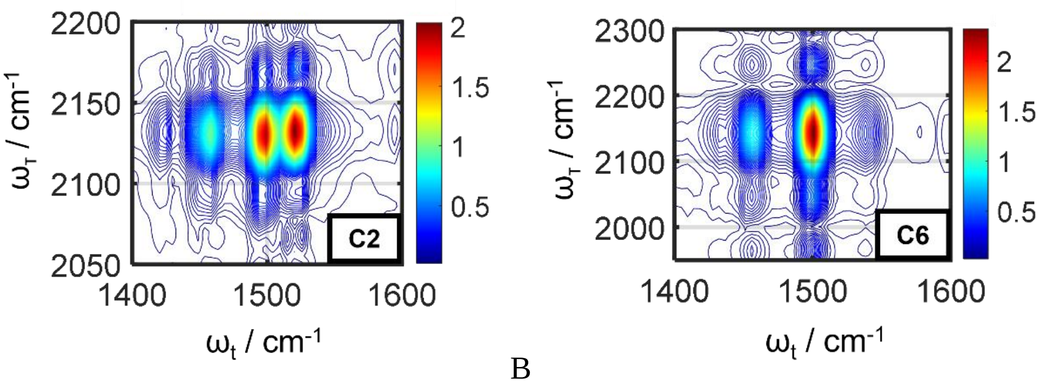

A B

Fig. S4. 2DIR magnitude spectra of (A) C2 at T = 3.5 ps and (B) C6 at T = 3.2 ps. Note that the peaks above and below the main line of peaks at ω_τ = 2130 cm^{-1} (C2) and 2140 cm^{-1} (C6) are mostly ringing peaks due to short τ scans.

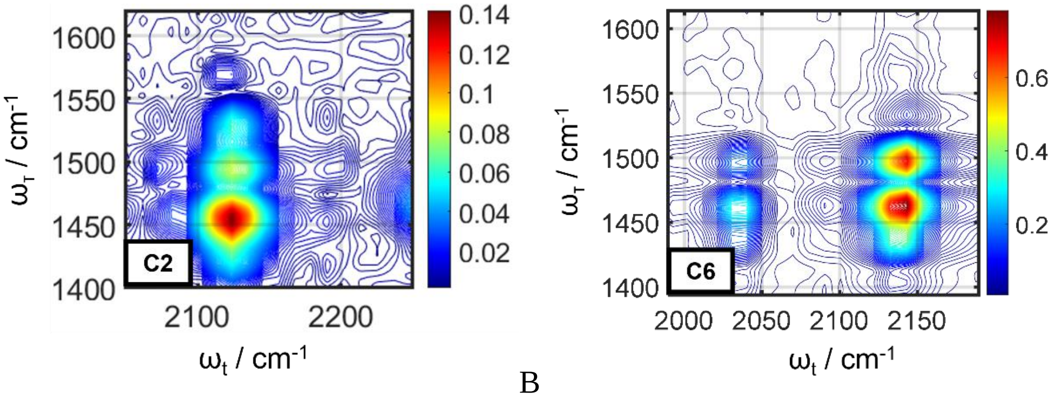

A B

Fig. S5. 2DIR magnitude spectra of (A) C2 at T = 3.5 ps and (B) C6 at T = 4.5 ps

4. Percent Contribution of Functional Groups to Normal Modes

The DFT-computed normal mode displacements along respective Cartesian axes, x_i, y_i, and z_i, are normalized according to Eq. S1.

$$\sum_i^{All}(x_i^2 + y_i^2 + z_i^2) = 1 \qquad \text{(S1)}$$

Here summation is performed over all atoms in the molecule. A percent contribution of a functional group to a normal was computed as

$$\%\, contribution = \sum_i^{Group}(x_i^2 + y_i^2 + z_i^2) \times 100\%, \qquad \text{(S2)}$$

where summation is performed over all atoms of the group.

5. Diagonal and Cross Peaks in the Fingerprint Region

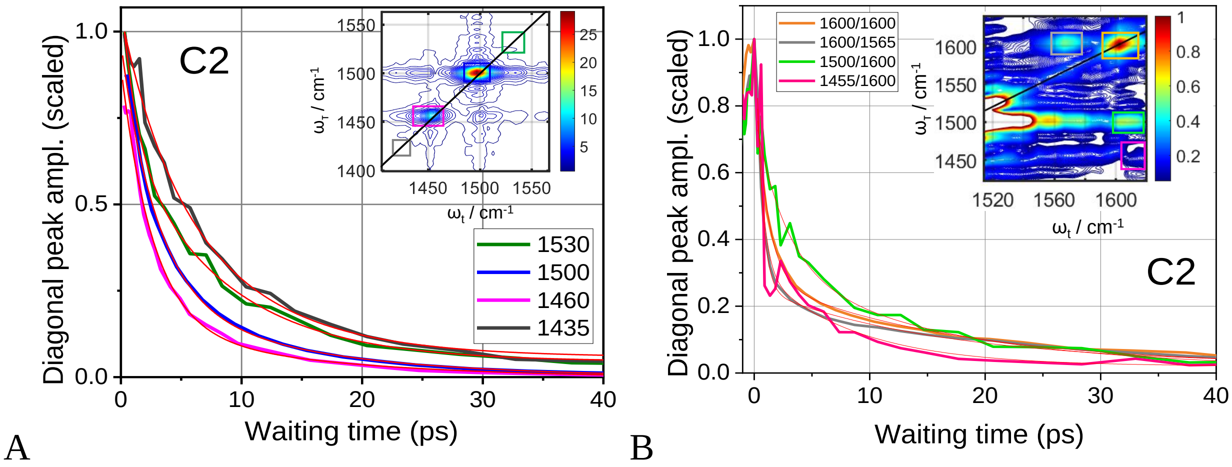

Fig. S6. Scaled waiting-time traces for indicated diagonal and cross peaks for C2. **A**. All peaks (1530, 1500, 1460 and 1435 cm^{-1}) were fitted globally with a double-exponential decay function (red lines). **B**. Cross peaks in the fingerprint region for C2 were analyzed and fitted with a double-exponential decay function (red lines) The insets in A and B show 2DIR spectra measured at 3.5 ps with color-matching boxes indicating the cross-peak integration regions.

6. Relaxation Channels of $\nu_{C\equiv C}$ for C6F

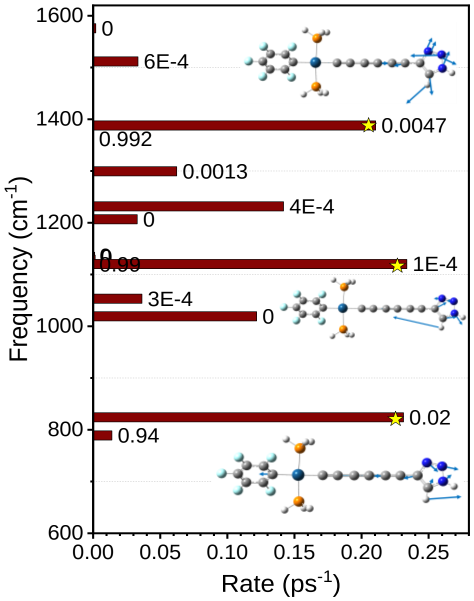

Figure S7. Rates of dominant relaxation channels of $\nu_{C\equiv C}$ computed for C6F. The displacements of the most contributing normal modes (labeled with stars) are shown as insets. Delocalization factors, χ, are shown for each normal mode to the right of its rate bar.

Figure S7 shows the dominant relaxation channels for the $\nu_{C\equiv C}$ tag in C6F, presented as rate bars populating various daughter states. Each relaxation daughter mode is labeled with a χ value (Fig. S7, right of bars), which represents the level of mode delocalization between the F_5Ph and C_n-Tri ligands across the Pt center (excluding motions at the PH_3 ligands). Similar to C2F, there is a negligible relaxation of the $\nu_{C\equiv C}$ tag directly into the F_5Ph localized modes.

7. Mode Delocalization in C6F

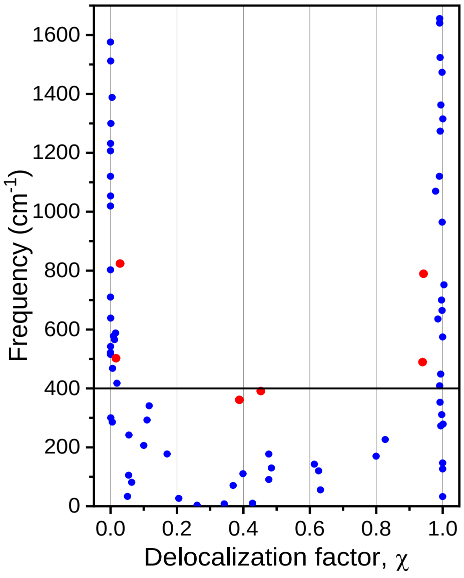

Figure S8. Delocalization factor, χ, for all normal modes below 1650 cm^{-1} in C6F. Three pairs of significantly delocalized high-frequency modes are shown with red circles.

8. Electrical Dipole-Dipole Coupling Across Pt Center

The through-space electrical coupling of the modes across the Pt center was computed using a point dipole approach. The electrical dipole-dipole coupling was computed using Eq. S4.

$$U_{12} = -k\frac{\mu_1^{\mathrm{tr}}\mu_2^{\mathrm{tr}}}{R^3} \qquad \text{(S4)}$$

Here μ_i^{tr} are the transition dipoles of the interacting modes, R is the distance between the oscillators, and k = 8.99 10^9 $C^{-2}Nm^2$. The transition dipole was calculated using the DFT-computed IR intensity by Eq. S5.[1]

$$A_a = \frac{N_A\,\pi\,(\mu_1^{\mathrm{tr}})^2}{3c^2}\left(\frac{\partial\vec{\mu}}{\partial Q_a}\right)^2 \qquad \text{(S5)}$$

Here A_a is the IR intensity in m/mol, N_A is Avogadro's number, c is the speed of light. The largest transition dipole moments of the pairs of coupled modes in C2 were found for the modes at 1362 and 1355 cm^{-1}, at 0.04 and 0.09 D, respectively. The interaction energy computed for the distance, R, of 5.3 Å is ca. 0.13 cm^{-1}. The largest IR intensity for C6 is found for the pair at 824 and 789 cm^{-1}, where the transition dipoles are 0.1 and 0.084 D, respectively, resulting in interaction energy of 0.08 cm^{-1} for R = 8 Å. The interaction energies for other coupled pairs for both C2 and C6 are not exceeding 0.05 cm^{-1}.

9. Comparison the Diagonal Peak Dynamics for C2 and C6

Figures S10-S12 show diagonal waiting-time traces for C2 and C6 peaks. Figure S9C,D,E and Figure S11 show multiple overlaying traces (scaled) measured in different experiments to demonstrate experimental reproducibility. Figure S9A,B,C and Figure S10 show traces for both C2 and C6 to indicate the similarities and differences of the traces for indicated diagonal peaks.

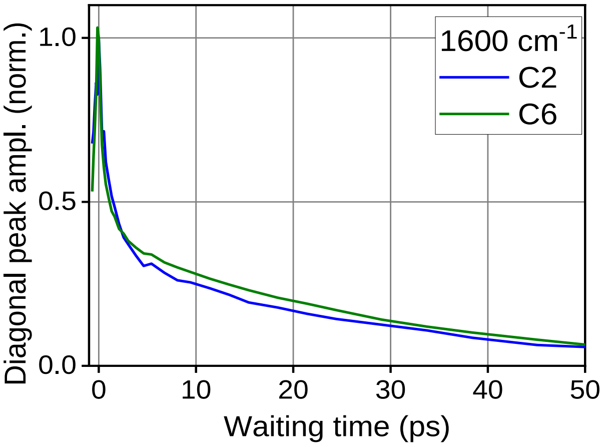

A

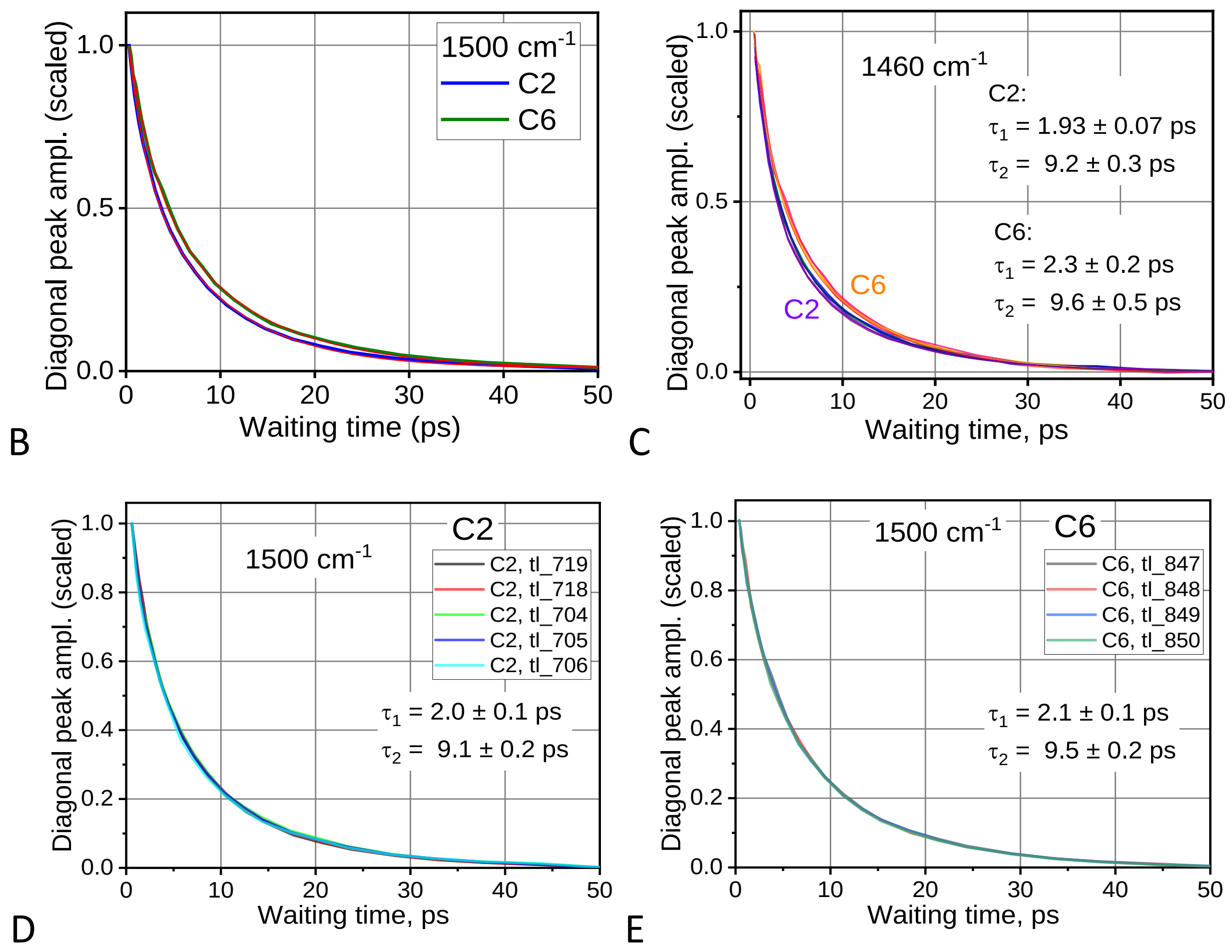

Figure S9. Comparison of scaled waiting-time dependences for C2 and C6 for diagonal peaks at (A) 1600, (B) 1500, and (C) 1460 cm^{-1}. Panels C-E show multiple overlaying traces measured in different experiments. The results of the fitting with a double-exponential function are shown in Table 4.

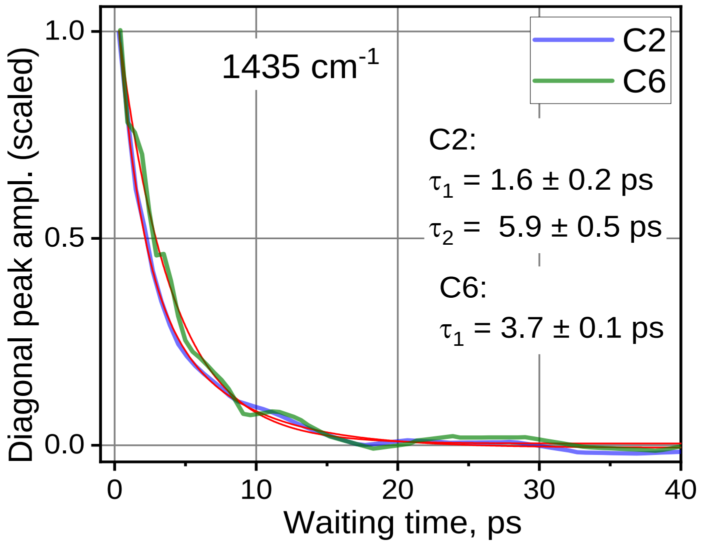

Figure S10. Waiting-time dependences for the diagonal peaks at 1435 cm^{-1} for C2 (blue lines) and C6 (red and cyan lines). Thin red lines show the fit results with a double exponential (C2) and single exponential (C6) functions. The 1435 cm^{-1} mode involves C-N stretching of the Tri ring and CH_2 scissoring motions.

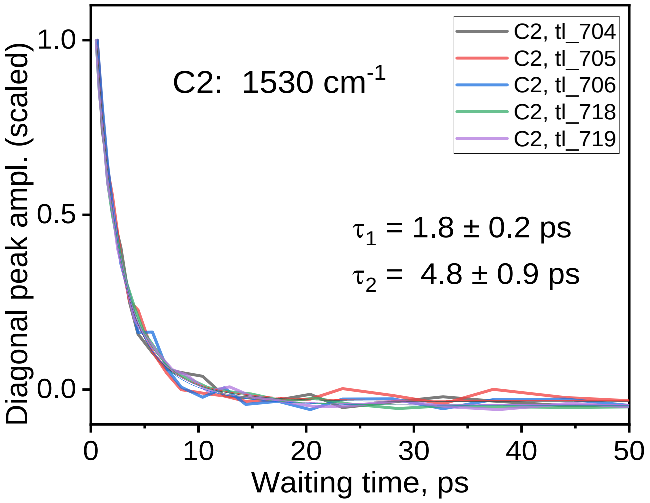

Figure S11. Comparison of five scaled waiting-time dependences for diagonal peaks at 1530 cm^{-1} for C2. Thin lines show the results of the global fit with a double-exponential function. The fit results are shown as inset. Note that the mode at 1530 cm^{-1} involves asymmetric stretching of the three carbon atoms, two of the Tri moiety and one of the alkyne linker. As a result, the mode frequency depends on the alkyne group length, observed at 1525 cm^{-1} in C2 and at 1540 cm^{-1} in C6.

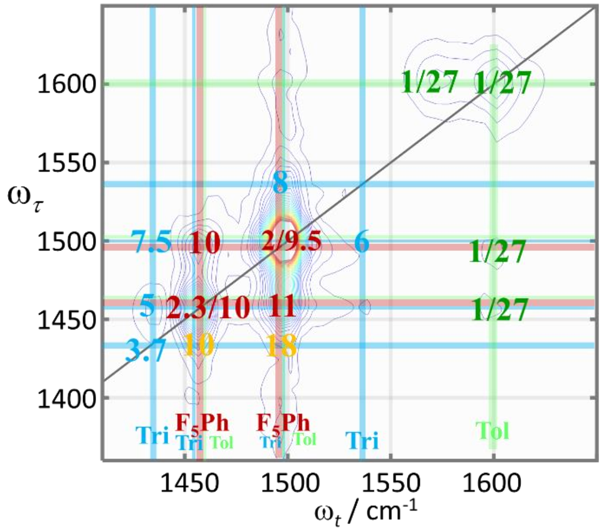

Figure S12. Exponential decay times measured are summarized for each diagonal and cross peak, also reported in Tables 4-6. The vertical and horizontal lines are color coded to indicate FTIR contributions originated from different ligands, F_5Ph (red), Tri (blue), and Tol (green).

Figure S12 summarizes graphically the exponential decay times measured for each diagonal and cross peak for C6.

References

1. Dybal, J.; Krimm, S., Dipole derivatives and infrared intensities of the ester group. An ab initio and force field study of methyl acetate. *J. Mol. Struct.* **1988,** *189*, 383-392.
